# A genome-scale mining strategy for recovering novel rapidly-evolving nuclear single-copy genes for addressing shallow-scale phylogenetics in *Hydrangea*

**DOI:** 10.1186/s12862-015-0416-z

**Published:** 2015-07-04

**Authors:** Carolina Granados Mendoza, Julia Naumann, Marie-Stéphanie Samain, Paul Goetghebeur, Yannick De Smet, Stefan Wanke

**Affiliations:** Department of Biology, Research Group Spermatophytes, Ghent University, K.L. Ledeganckstraat 35, 9000 Ghent, Belgium; Departamento de Botánica, Instituto de Biología, Universidad Nacional Autónoma de México, Apartado Postal 70-367, 04510 Coyoacán, Distrito Federal Mexico; Institut für Botanik, Technische Universität Dresden, Zellescher Weg 20b, 01062 Dresden, Germany; Instituto de Ecología, A.C., Centro Regional del Bajío, Avenida Lázaro Cárdenas 253, 61600 Pátzcuaro, Michoacán Mexico

**Keywords:** Data mining, Fine-scale phylogenetics, *Hydrangea* sect. *Cornidia*, Phylogenetic signal, Phylogenetic noise

## Abstract

**Background:**

Identifying orthologous molecular markers that potentially resolve relationships at and below species level has been a major challenge in molecular phylogenetics over the past decade. Non-coding regions of nuclear low- or single-copy markers are a vast and promising source of data providing information for shallow-scale phylogenetics. Taking advantage of public transcriptome data from the One Thousand Plant Project (1KP), we developed a genome-scale mining strategy for recovering potentially orthologous single-copy markers to address low-scale phylogenetics. Our marker design targeted the amplification of intron-rich nuclear single-copy regions from genomic DNA. As a case study we used *Hydrangea* section *Cornidia*, one of the most recently diverged lineages within Hydrangeaceae (Cornales), for comparing the performance of three of these nuclear markers to other “fast” evolving plastid markers.

**Results:**

Our data mining and filtering process retrieved 73 putative nuclear single-copy genes which are potentially useful for resolving phylogenetic relationships at a range of divergence depths within Cornales. The three assessed nuclear markers showed considerably more phylogenetic signal for shallow evolutionary depths than conventional plastid markers. Phylogenetic signal in plastid markers increased less markedly towards deeper evolutionary divergences. Potential phylogenetic noise introduced by nuclear markers was lower than their respective phylogenetic signal across all evolutionary depths. In contrast, plastid markers showed higher probabilities for introducing phylogenetic noise than signal at the deepest evolutionary divergences within the tribe Hydrangeeae (Hydrangeaceae).

**Conclusions:**

While nuclear single-copy markers are highly informative for shallow evolutionary depths without introducing phylogenetic noise, plastid markers might be more appropriate for resolving deeper-level divergences such as the backbone relationships of the Hydrangeaceae family and deeper, at which non-coding parts of nuclear markers could potentially introduce noise due to elevated rates of evolution. The herein developed and demonstrated transcriptome based mining strategy has a great potential for the design of novel and highly informative nuclear markers for a range of plant groups and evolutionary scales.

**Electronic supplementary material:**

The online version of this article (doi:10.1186/s12862-015-0416-z) contains supplementary material, which is available to authorized users.

## Background

Identification of informative markers for specific phylogenetic questions finds one of its major challenges at shallow-scale phylogenetics, mostly along the gradient between species and population level. In particular, short internodes in phylogenetic trees, where lineages split in quick succession, are difficult to resolve [1]. Plastid and nuclear ribosomal DNA loci (nrDNA) have predominantly been used in phylogenetic studies of flowering plants [2–7]. However, their applicability for resolving shallow divergences is limited by several characteristics. Plastid markers show reduced sequence divergence at a low phylogenetic level in most plant lineages due to the slow and nearly constant rate of plastome evolution relative to the nuclear genome [3, 8]. When used as the sole source of information, plastid markers provide only one side of the information about processes such as hybridization and introgression [9, 10], since they are inherited maternally in most angiosperms [3]. Nuclear ribosomal DNA markers occur in high numbers of copies, causing problems for assessing orthology [4, 11]. Furthermore, due to incomplete homogenization of nrDNA gene families (i.e. partial concerted evolution; [12, 13]), biparental inheritance might be traced inconsistently in hybrids [3, 10, 14, 15].

Nuclear single or low-copy genes, and particularly their introns, have the potential to overcome these disadvantages. Compared to plastid markers, they are generally more variable, potentially providing a higher number of parsimony informative characters per sequenced bases [9, 14]. The biparental inheritance of nuclear markers facilitates the identification of possible hybridization and polyploidization events, and when used in concert with plastid markers the directionally of hybridization can be inferred [9, 10, 14–19]. When present in low- or single-copy, nuclear markers are a highly valuable source of putative orthologous loci, that are increasingly being accessed in plant phylogenomics [7, 9, 10, 15, 19–34]. For a long time in plant phylogenetics, data and technology were unavailable or at least limited to explore and broadly screen the nuclear genome. One of the first genome scale comparative approaches identified a set of 959 nuclear single-copy genes (NSCG) shared by *Arabidopsis thaliana*, *Populus trichocarpa*, *Vitis vinifera* and *Oryza sativa* (APVO SSC genes; [14]). The utility of these loci has been demonstrated by reconstructing angiosperm phylogenetic relationships from deep to shallower evolutionary depths [9, 14, 24].

Compared to the functionally constrained exons, the introns of the APVO SSC genes provide additional variability shown to be useful at and below species level [9]. Even though the benefits of employing potential nuclear single-copy genes (NSCG) for plant phylogenetics were highlighted years ago [2, 3, 35], only now are they becoming more popular and widely used with the expansion of nuclear genome resources. Next Generation Sequencing Technologies (NGS) generate an increasing amount of genomic resources [31, 36]. This coupled with the recent proliferation of novel genome-partitioning strategies have considerably facilitated the application of NSCG to plant phylogenetic studies [37]. Two highly promising genome partitioning strategies currently applied to plant phylogenetics are anchored hybrid enrichment, currently under test and development, (Alan Lemmon, Florida State University, USA, personal communication) and transcriptome sequencing. Among these two strategies, mining publicly available transcriptome resources for developing novel NSCG markers for non-model plant species is being increasingly applied [29, 38–41]. Recent approaches for the discovery of novel NSCG markers from public or newly produced transcriptome data range from the application of elaborate bioinformatics pipelines [29, 39, 40] to the development of automated tools (e.g. MarkerMiner; [42]).

International projects, such as the “1000 plants” (1KP, http://www.onekp.com) use more cost efficient strategies such as transcriptome sequencing, compared to whole genome sequencing, and continuously make nuclear genome-scale information accessible for an increasing number of green plants. The present study benefits from these transcriptome resources for identifying novel rapidly-evolving nuclear loci for shallow-scale phylogenetics in *H.* sect. *Cornidia*. This section of mostly Neotropical species is one of the most recently diverged lineages within the tribe Hydrangeeae (Hydrangeaceae, Cornales; [43–45]) and is composed of 13 currently accepted species and a yet unknown number of new species of evergreen, root-climbing hortensias [46]. Although resolution and support for the internal relationships of *Hydrangea* have progressively been improved by the addition of highly variable coding and non-coding plastid regions [44, 45] and nuclear ribosomal ITS sequences [43], a number of phylogenetic splits at the species level and below remain unresolved. Previously used plastid markers have shown extremely low variability among studied *H*. sect. *Cornidia* representatives. This has been attributed to a potential rapid or recent diversification event [44] and could additionally be due to a very slow rate of sexual reproduction. Nuclear single or low-copy sequence data have been generated for only two Hydrangeaceae species in the past, namely *Philadelphus incanus* (*SMC1*, *SMC2*, *MSH1*, *MLH1* and *MCM5* genes, [13]) and *H. paniculata* (Xdh gene, [47]). However, none of these nuclear loci have been employed for a wider taxonomical sampling in the family. All these lines of evidence make *H*. sect. *Cornidia* an excellent study case for testing the performance of potential NSCG retrieved by a genome scale mining strategy for resolving fine scale phylogenetic relationships.

Methods such as those developed by Townsend [48] and Townsend et al. [49] provide an efficient approach for estimating and comparing the utility of different molecular markers for specific phylogenetic questions. Townsend’s phylogenetic informativeness (PI) method provides a quantitative measure of the phylogenetic signal in markers by quantifying the probability that a character changes at a certain position of a tree, and does not undergo further change [48]. For this, the evolutionary changes across sites are compared against an ideal change rate based on a reference ultrametric tree, where branches are proportional to evolutionary units. The PI method has been implemented in the freely-accessible online program PhyDesign [50]. This program requires a priori sequence data for the candidate loci and only one reference tree that can, for instance, be calculated from a subset of the taxa of interest. Although the PI method does not account for potential phylogenetic noise, Townsend and collaborators [49] recently proposed an analytical solution for estimating the probabilities of correct, incorrect and polytomous resolutions given rates of evolution and a defined state space.

Taking advantage of these recently published analytical tools, as well as of public genome resources, our aims are to: 1) develop a genome-scale mining strategy for recovering novel, intron-rich, nuclear single-copy loci potentially useful for shallow-level phylogenetics in *Hydrangea*, 2) design a set of primers for successfully amplifying and sequencing these regions from genomic DNA in *Hydrangea*, 3) test the success of some of these markers in resolving shallow divergences in *H*. sect. *Cornidia*, 4) estimate the phylogenetic informativeness of the selected nuclear markers and compare them with other previously used “fast” evolving plastid markers: the *rpl32–ndhF* intergenic spacer (IGS), *trnV–ndhC* IGS, *trnL–rpl32* IGS and *ndhA* intron, and 5) compare the performance of nuclear and plastid markers for resolving shallow and deeper-level divergences in *Hydrangea*.

## Results

### Mined potential NSCG

**Fig. 1:**
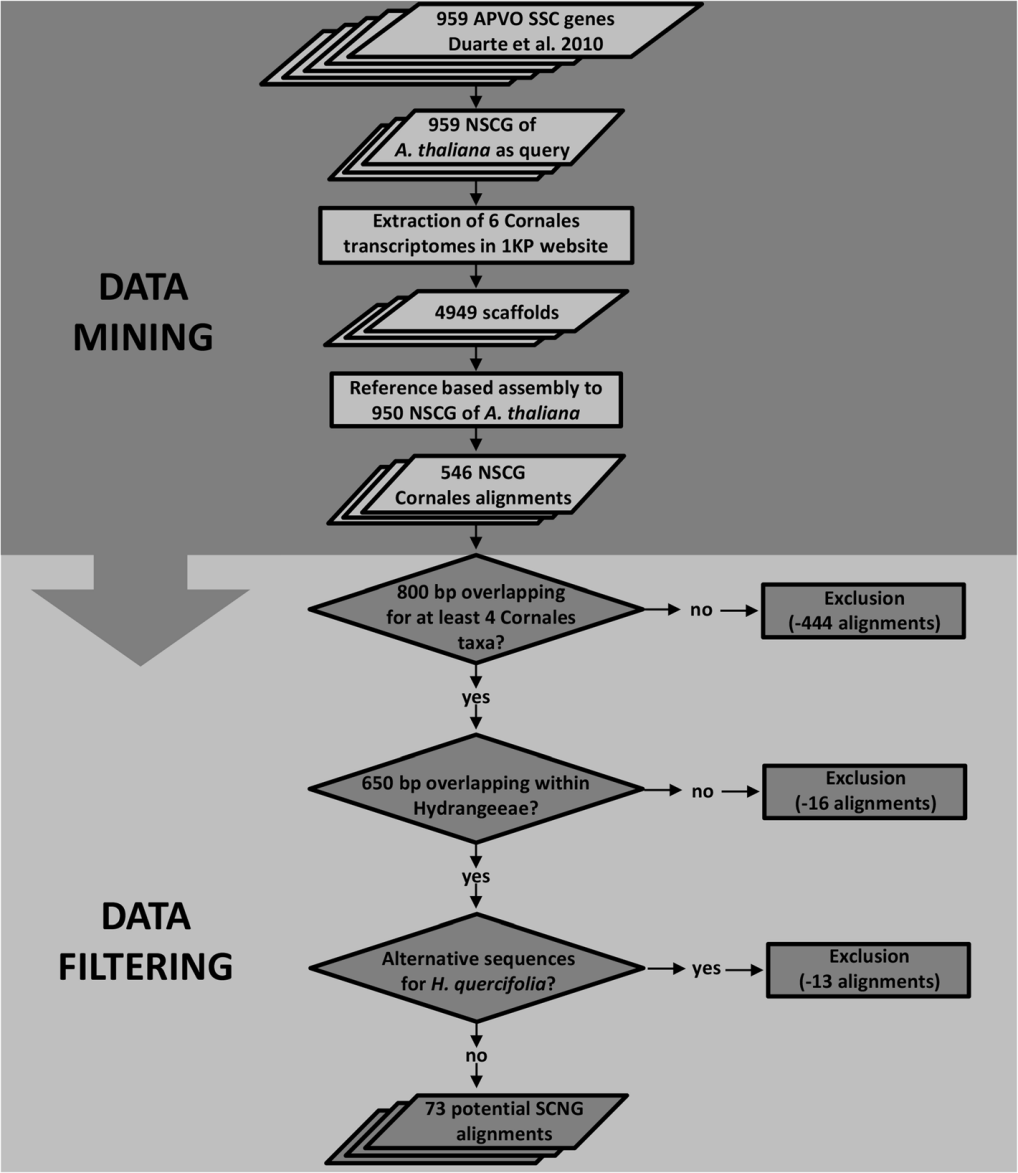
Nuclear single-copy gene mining strategy and data filtering. Data mining and filtering strategy employed for identifying potential NSCG for resolving shallow evolutionary divergences in *H*. sect. *Cornidia*

**Fig. 2:**
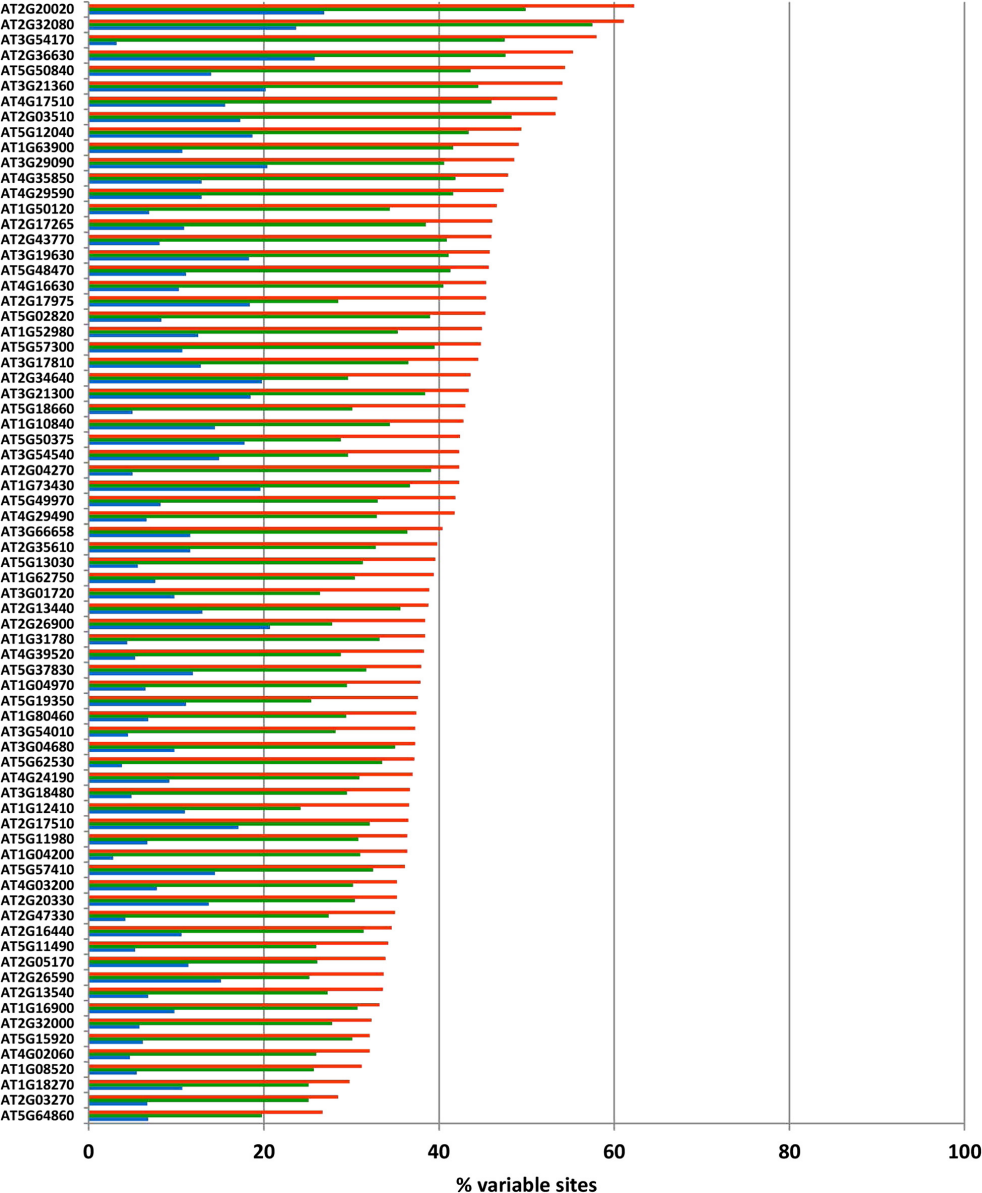
Retained potential NSCG after the entire data mining and filtering process. Genes have been ordered by their percent of variability among Cornales representatives. Orange bars denote the percent of variable sites among the six Cornales taxa, whereas green and blue bars represent the percent of variable sites among the four Hydrangeaceae representatives and the two tribe Hydrangeeae taxa, respectively

Our data mining process retrieved 4949 Cornales scaffolds that could be aligned to 546 *A. thaliana* single-copy gene orthologs. This pool of genes was used to select the most promising and the easiest to handle markers for *H.* sect. *Cornidia*. We were aiming for gene alignments where a major part of the gene was covered by at least four of the 1KP Cornales species. This is crucial for estimating phylogenetic utility of the candidate markers, as well as for successful primer design. Among the 546 initial alignments, 444 showed low coverage of Cornales representatives and additional 16 showed low coverage of the two Hydrangeeae taxa (Fig. 1). Therefore these alignments were not considered any further. Multiple scaffold sequences were detected for *H. quercifolia* in 13 of the remaining alignments and were therefore excluded from further steps. A total of 73 potential NSCG were retained after filtering (Fig. 1). From these alignments, 14 contained the six Cornales representatives, whereas 53 lacked either *Curtisia dentata* or *Deutzia scabra* and six did not have either *Philadelphus inodorus* and *Curtisia dentata*, or *Curtisia dentata* and *Deutzia scabra*. When considering transcriptome sequences from all Cornales taxa, alignments showed 26.7-62.3 % variable sites (Fig. 2). However, the range of variable sites was lowered to 19.8-57.5 % when only the four Hydrangeaceae representatives were considered and to 2.8-26.9 % between the two representatives of tribe Hydrangeeae (Fig. 2).

### Amplification and sequencing of selected NSCG

**Fig. 3:**
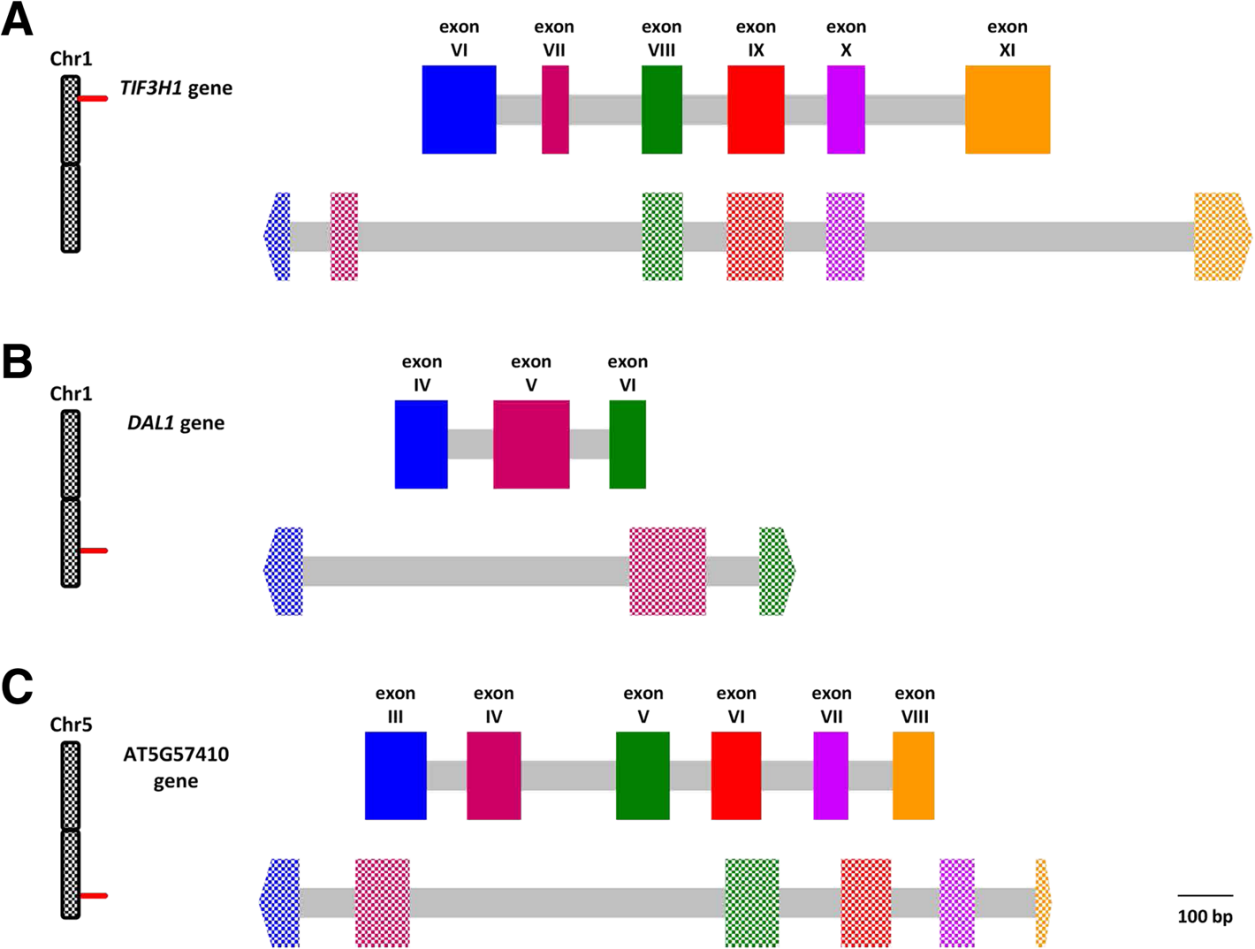
Nuclear single-copy gene models. Models for the amplified portions of the *TIF3H1* (**a**), *DAL1* (**b**), and AT5G57410 (**c**) genes used for resolving shallow evolutionary divergences in *H*. sect. *Cornidia*. Lengths of the respective regions are drawn as found in *H. seemannii* (Granados M. et al. 479, Pueblo Nuevo, Durango, Mexico). Solid colored large boxes identify exon regions of *A. thaliana*, whereas dotted colored large boxes denote exon regions of *H. seemannii*. Homology of exon regions between the two species is indicated by common colors. Non-coding regions are indicated by grey boxes. Relative position of each gene within the respective *A. thaliana* chromosome is shown as indicated in TAIR (http://www.arabidopsis.org/)

From the 27 tested primer pairs, 11 yielded amplicons with single bands for the reduced taxon sampling. The remaining 16 primer combinations either produced multiple bands or did not amplify in either *H*. sect. *Cornidia* or the outgroup taxa. Primer pairs producing multiple PCR products corresponded to the AT2G17975, AT5G48470, AT3G54170 and AT5G12040 *A. thaliana* orthologs. Among the regions that amplified a single band, only three produced clear sequences, and were therefore selected for further tests using the extended taxon sampling. The amplified regions correspond to portions of the *A. thaliana* gene orthologs AT1G10840, AT1G63900 and AT5G57410 (Fig. 3). According to TAIR (http://www.arabidopsis.org/), the AT1G10840 ortholog corresponds to the gene *TIF3H1* (Translation initiation factor 3 subunit H1) encoding the eukaryotic initiation factor 3H1 subunit and is part of the eukaryotic translation initiation factor 3 (eIF-3) complex. The AT1G63900 gene, known as the *DAL1* (DIAP1-like protein 1) or *SP1* (Suppressor of PPI1 locus 1) gene (hereafter referred to as *DAL1* gene) encodes a RING-type ubiquitin E3 ligase present in the chloroplast outer membrane. The AT5G57410 ortholog is known to encode a microtubule-associated protein. Gene Ontology annotations [51] for these genes are provided in Additional file 1. A region of ca. 430 bp located in the 5′ portion of the amplified *DAL1* gene region was excluded from the final alignments because long mononucleotide repeats yielded low quality sequences. Compared to *A. thaliana*, coding regions in *H*. sect. *Cornidia* species show high sequence similarity, while non-coding regions were overall considerably greater in length (Fig. 3).

### *Phylogenetic relationships* of Hydrangea *sect.* Cornidia

**Fig. 4:**
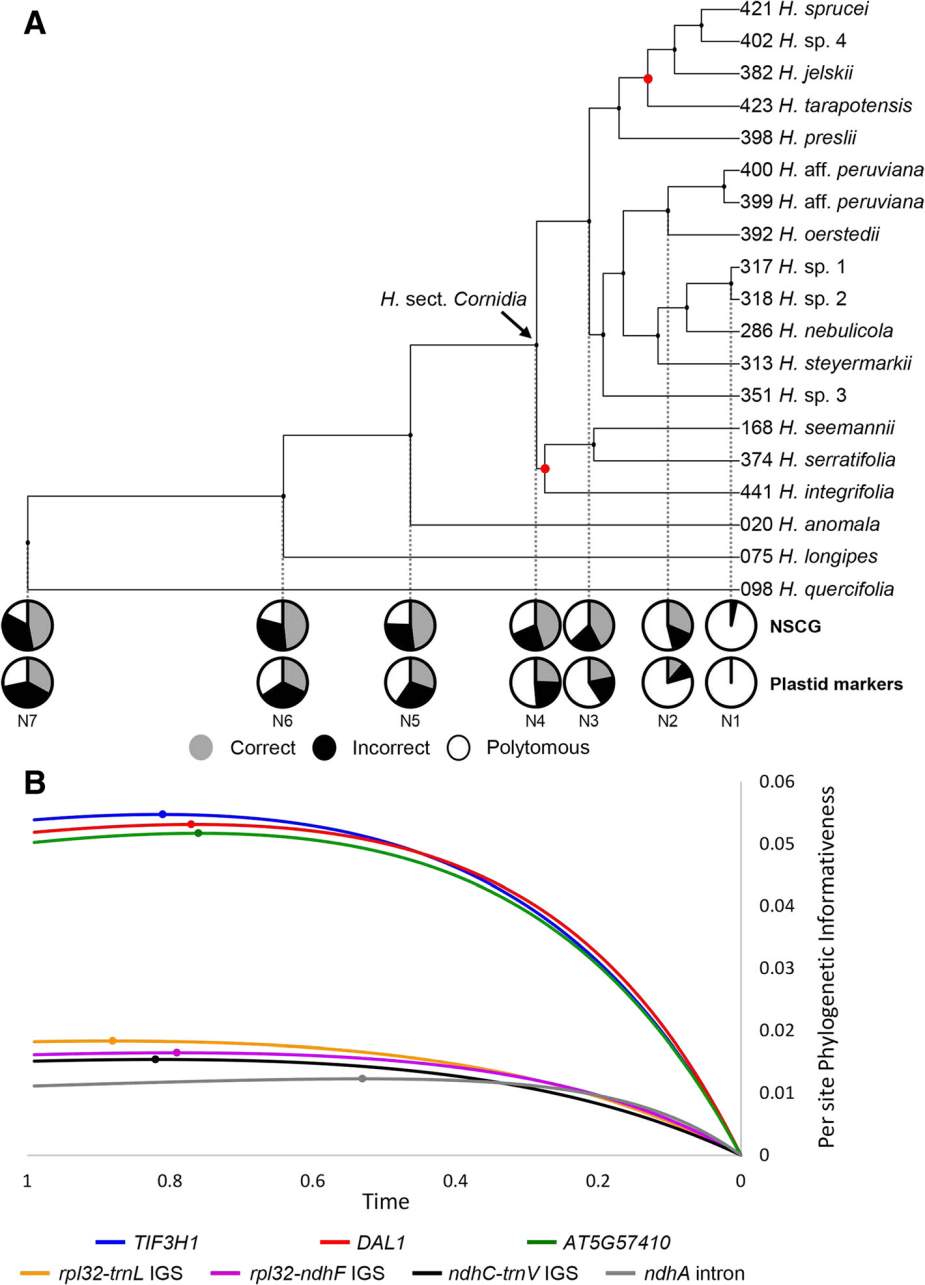
Phylogenetic informativeness, signal and noise of nuclear and plastid markers. Pie charts show the relative probabilities of correct (grey), incorrect (black) and polytomous (white) resolution of nuclear and plastid datasets across a range of individual nodes (N1-N7) within the phylogeny of *H*. sect. *Cornidia* and its close relatives. The probability of nuclear markers recovering incorrect resolutions is lower than their respective probability of recovering correct resolutions across all nodes. In contrast, plastid markers show higher probabilities of recovering incorrect than correct resolutions at the deepest nodes (N5 to N7; **a**). Per site PI across time of the individual nuclear and plastid regions (**b**). Red circles indicate BS <85 for internal nodes

Our combined nuclear and plastid data set comprised a total of 7994 characters. Due to uncertain homology assessment, 357 characters were removed from subsequent analyses. Positions of excluded regions in the initial alignment, as well as important characteristics from each nuclear and plastid data subsets are shown in Additional file 2. With the exception of two internal nodes indicated below, our total combined nuclear and plastid ML analysis retrieved strongly supported clades with BS ≥ 85 (Fig. 4). No statistically supported incongruences were detected between the combined plastid and combined nuclear analyses. However, the nodal support based on the combined nuclear dataset was remarkably higher than that retrieved with the combined plastid matrix (Additional file 3).

*Hydrangea* sect. *Cornidia* was recovered as monophyletic with *H. anomala* of section *Calyptranthe* as its sister species. Within *H*. sect. *Cornidia* three main clades were recovered. The first of them (BS = 56) consisting of (*H. integrifolia* (*H. seemannii* + *H. serratifolia*)) is sister to a lineage containing the other two main clades. In the second main clade an undescribed species from Costa Rica (*H*. sp. 3) is sister to a clade that further divides into two groups. The first of these groups includes a grade of *H. steyermarkii, H. nebulicola* and two undescribed Mexican species (*H*. sp. 1 and *H*. sp. 2), while the second is formed by *H. oerstedii* sister to *H*. aff. *peruviana* (Fig. 4). The third main clade consists of a grade of *H. preslii*, *H. tarapotensis*, *H. jelskii* and *H. sprucei* plus an undescribed Ecuadorian species (*H*. sp. 4). The phylogenetic position of *H. tarapotensis*, however, received low statistical support (BS = 65).

### Phylogenetic informativeness of NSCG and plastid markers

Per site PI of NSCGs was up to threefold higher than that of the plastid markers *rpl32–ndhF* IGS, *trnV–ndhC* IGS, *trnL–rpl32* IGS and *ndhA* intron (Fig. 4). The three NSCGs showed highly similar per site PI profiles with a steep increase during the shallowest divergences within *H*. sect. *Cornidia* and followed by a more steady increase reaching their PI_max_ at the times 0.76 (AT5G57410), 0.77 (DAL1) and 0.81 (TIF3H1; Fig. 4). Lower, rather flat profile curves were recovered for the four plastid regions (Fig. 4). The curves of the plastid markers show a moderate initial increase and a flatter shape reaching their PI_max_ at the times 0.79 (*rpl32-ndhF* IGS), 0.82 (*ndhC-trnV* IGS) and 0.88 (*rpl32-trnL* IGS; Fig. 4). Only the *ndhA* intron reached its PI_max_ considerably earlier (at time 0.53) than any other plastid or nuclear marker, leading to the lowest per site PI values (Fig. 4).

### Performance of nuclear and plastid markers in resolving shallow and deeper-level divergences

The phylogenetic signal and noise analysis was applied to assess the probability of the nuclear and plastid data sets resolving shallow and deeper-level divergences. As compared to our reference tree resulting from the combined analysis of all nuclear and plastid markers uploaded in PhyDesign, nuclear single copy genes retrieved considerably higher probabilities of correct over incorrect resolutions for shallow nodes; these probabilities become more similar towards deeper nodes (Fig. 4). In contrast, plastid markers recovered similar probabilities of correct and incorrect resolutions from the shallowest nodes and lower probabilities of correct resolution relative to incorrect resolution at the two deepest nodes (Fig. 4). The probability of polytomous resolution was considerably higher for the plastid relative to the nuclear data set across all nodes.

## Discussion

### Genome-scale mining strategy

Large amounts of publicly available genome-scale data from a broad variety of green plants offer new and promising prospects in plant systematics. The constantly increasing public resources of nuclear sequence data provide a vast pool for nuclear marker development. It is now possible to identify orthologous nuclear markers for nearly any plant group of interest and for a wide range of phylogenetic levels. The transcriptome mining approach described here for Hydrangeaceae is highly efficient and powerful for identifying novel nuclear single-copy genes informative at shallow evolutionary depths. A pool consisting of hundreds of nuclear candidate genes can quickly be reduced to a set of best and most efficient markers for a specific phylogenetic question following a few simple criteria. Specifically, we targeted orthologous genes with intron-rich regions that can be amplified broadly and sequenced from genomic DNA. A high number of initial candidate genes allowed the efficient discarding of less promising phylogenetic markers and still left a good number of potentially orthologous, highly informative nuclear loci.

Among the excluded nuclear genes, we identified a handful with different sequence variants for *H. quercifolia*, suggesting multiples copies for these nuclear genes in this species and potentially in other *Hydrangea* species. Several factors can explain the observed potential gene duplications. First, all flowering plants are ancestral polyploids [52] and polyploidization events are common among angiosperms lineages [34, 53, 54]. In addition to the gamma polyploidization, predating the diversification of the core Eudicots, three additional polyploidization events are known to have occurred in the Asterid clade according to genome wide comparisons [55, 56]. Second, alterations to the basic chromosome number in *Hydrangea* (2n = 36) are known as a result of descending dysploidy in the *H. aspera* complex (2n = 30 and [34, 57, 58]). Since descending dysploidy is frequently generated by chromosome rearrangements such as centromere losses in monocentric chromosomes resulting in chromosome number reduction [59], little effect could be expected on the number of copies of a gene (gene dosage) according to Duarte et al. [14]. However, descending dysploidy is known to frequently follow polyploidy events [59], which in this case could have preceded the diversification of the genus. A specific polyploidy event predating the diversification of *Hydrangea* could explain the multicopy nature of some APVO genes [14] in *H. quercifolia* (2n = 2x = 36; [57]). Previously reported cases of triploids (*H. macrophylla*), tetraploids (*H. liukiuensis*), hexaploids (*D. febrifuga*), octoploids and decaploids (*H. yayeyamensis*; [60, 61]) sustain the notion of polyploidy being a frequent evolutionary event in the hortensias and their relatives.

Due to the large number of initial candidate genes, discarding those with multiple variants, did not limit our power for answering our phylogenetic question. However, researchers working with a limited amount of candidate markers may opt for determining the origin of such sequence variants (e.g. occurrence of alternative splicing of pre-mRNA molecules). If the observed sequence variants are not discovered at the genomic DNA level, they might be splice variants, and the specific marker could be retained. Otherwise, multiple genomic variants indicate gene duplication and the marker should be excluded from further phylogenetic analyses. For this reason, a large repertory of initial candidate genes is highly beneficial.

Another important advantage of our mining strategy is its broad applicability. The 73 potential NSCG could potentially be useful for other Cornales families since the genome-scale mining strategy implemented here included representatives from two other Cornales lineages. Additionally, our taxon sampling could easily be expanded by adding further lineages from outside Cornales. Our markers are likely useful at a range of taxonomic levels due to the observed exon variability. Moreover, our data mining and filtering strategy can be applied to any other plant group with enough genome resources. Hence, because of its versatility, this method has the potential to be used by a wide range of plant evolutionary biologists.

### Establishment of selected nuclear single-copy markers

The next relevant step in marker development, after potential candidates have been selected, is to screen for successful primer combinations and to test their applicability. A number of tested primer pairs did not yield PCR products. These primer combinations were designed based on exon sequences of six Cornales representatives including *H. quercifolia*. Since the latter species was included in our test taxon sampling, it is unlikely that amplification failure is due to the primer design. A more plausible explanation could be differences between *A. thaliana* and *H*. sect. *Cornidia* intron sizes. While exon size is highly conserved, intron size is considerably greater in *H*. sect. *Cornidia* relative to *A. thaliana*. These observations are in agreement with the 1C DNA content of *A. thaliana* and *Hydrangea* diploids which range from 0.05-0.23 pg and 0.98-3.5 pg, respectively (Plant DNA C-values Database, release 6.0, December 2012, http://data.kew.org/cvalues/). The differences in intron size and 1C DNA content suggest that a number of primer pairs fail to amplify because they likely spanned long introns that could not be amplified using regular PCR conditions.

Primer pairs belonging to four different genes were found to amplify multiple, two to four, PCR products of different sizes in *H. quercifolia* and one or more species from our test taxon sampling. These multiple PCR products could be the result of the amplification of more than one gene region due to low primer specificity, as well as the amplification of a multicopy gene region. In neither of these cases the primer pairs could be considered as good candidates, hence we excluded them from further tests.

Among retained potential single-copy candidate markers, three regions amplified a single band and produced clear sequence reads. The majority of the other regions showed multiple peaks for a nucleotide in the chromatograms, most likely as a result of multiple copies of similar size or differences among alleles, or long mononucleotide repeat units followed by a decrease in sequence quality. Sequence regions divided by highly repetitive elements, such as long mononucleotide repeats, are hard to bridge during sequencing and, if “successfully” sequenced, the number of repetitive elements is always uncertain, even when using certain next generation sequencing approaches (e.g. 454 pyrosequencing; [62]). Therefore, regions containing highly repetitive motifs are preferably removed from marker selection processes [63].

### *Phylogenetic utility of nuclear single copy genes for the case of* H*. sect.* Cornidia

Our total combined plastid and nuclear analysis obtained a fully resolved and highly supported phylogenetic hypothesis, with the exception of the affinities of *H. integrifolia* and *H. tarapotensis. Hydrangea integrifolia* is connected to the rest of the *H*. sect. *Cornidia* species by a short and deep internode. Such internodes are a common pitfall in plant phylogenetics [64] and generally require the inclusion of multiple markers that are highly informative for that specific divergence time scale [1, 24, 45]. A long branch leads to *H. integrifolia*, as well as each of the species *H. seemannii* and *H. serratifolia* —here recovered as closely related. These long branches are potentially the result of accelerated evolutionary rates due to relaxed evolutionary constraint of the non-coding regions (i.e. introns), which could promote sequence saturation and result in long-branch attraction among these three species (see [65]). Future studies should, however, include a more detailed data partitioning scheme that separates non-coding from coding regions, as well as 1st, 2nd and 3rd codon positions among coding regions, since it has been shown that moderate to high rates of substitution in coding regions do not always negatively affect phylogenetic reconstruction [66]. Inclusion of more taxa has been shown to break long branches [67]. Addition of other *H*. sect. *Cornidia* species might help reduce the potential long-branch attraction effect of *H. integrifolia* and help elucidate its affinities. Specifically, in the case of *H. tarapotensis,* internode and branch length does not seem to explain its unsupported affinities and none of the plastid and nuclear data recovered strongly supported relationships for this species. Inclusion of more informative data near the specific time scale of divergence of *H. tarapotensis* may help clarify its phylogenetic relationships.

Plastid and nuclear data did not show strongly supported incongruence justifying the viability of a total combined analysis. Both plastid and nuclear data retrieved strong support for the deepest divergences; however, nodal support for shallower divergences retrieved from the combined nuclear analysis surpassed by far that from the combined plastid regions. The latter suggests that the nodal support obtained for the low-level divergences was provided by phylogenetic signal mostly contained in the nuclear data.

### Phylogenetic informativeness of NSCG and plastid markers

Per site PI profiles of the nuclear markers were in general much higher than those of plastid markers. To our knowledge, no previous study has compared PI profiles of nuclear single-copy and plastid data. From our comparison we can observe considerably higher phylogenetic utility of nuclear single-copy genes than plastid markers at most of the evolutionary depths of our phylogenetic framework. All nuclear and plastid markers peaked deeper than the time of divergence of *H*. sect. *Cornidia*, suggesting that little or low phylogenetic noise can be expected along the evolutionary time scale of *H*. sect. *Cornidia*. At deeper taxonomical levels, potential phylogenetic noise, as indirectly inferred from PI profiles, can be expected from the *ndhA* intron around the divergence time of *H. anomala,* and from the rest of the markers close to the divergence time of *H. longipes*. Special attention should be paid to the *ndhA* intron since despite its low variability, this plastid IGS peaked even earlier than the more variable nuclear markers. A combined plastid and nuclear analysis excluding the *ndhA* intron (Additional file 3) retrieved the same phylogenetic relationships as our total combined plastid and nuclear analysis; however, the bootstrap support for the sister relationship of *H. nebulicola* and the two undescribed Mexican species increased from 86 to 93. The latter suggests that the *ndhA* intron not only provides low phylogenetic signal at shallow evolutionary depths, but also potential phylogenetic noise for deeper divergences in *Hydrangea*.

### Ability of NSCG and plastid markers for resolving shallow and deeper-level divergences

A recent angiosperm-wide study by Han et al. [53] found that the addition of single-copy gene introns resulted in increased branch support, although it also uncovered notable incongruences. Based on this finding, Han et al. [53] recommended the use of single-copy intron sequences for lower taxonomic levels. Intron variability within our nuclear data set also seems to present the appropriate evolutionary pace for resolving shallow divergences in *H.* sect. *Cornidia* without introducing phylogenetic noise. Plastid markers might be more appropriate for deeper taxonomic levels, at which non-coding regions of nuclear markers could potentially introduce phylogenetic noise. Future studies implementing highly efficient techniques for the collection of high‐throughput sequencing data (e.g. [63]) should consider analyzing these larger data sets under a scheme that accounts for possible gene-tree incongruences and phylogenetic noise potentially introduced by numerous fast-evolving sites [68].

## Conclusion

The genome-scale mining strategy developed and demonstrated here successfully recovered a number of novel, rapidly-evolving nuclear single-copy genes useful at different taxonomic depths not only within *Hydrangea*, but with potential for use in other Cornales taxa and beyond. Other transcriptome mining strategies such as that developed by Rothfels et al. [29], Tonnabel et al. [39] and Pillon et al. [40] confirm the great potential of these approaches for developing highly informative markers for phylogenetic reconstructions of different plant lineages and at a range of taxonomic depths. The automation of such transcriptome mining procedures by Chamala et al. [42] certainly represents a valuable contribution to the plant evolutionary research community, allowing the application of these strategies for researchers with little knowledge on bioinformatics. The 1KP plant transcriptomes are an invaluable source of information for developing orthologous nuclear markers that can complement established conventional markers and help resolve currently unknown evolutionary relationships among green plants. The application of other highly efficient genome partitioning strategies such as the anchored hybrid enrichment, which can potentially allow for the recovery of hundreds of orthologous single copy nuclear loci, is another exciting and promising possibility for upcoming plant phylogenetic studies.

## Methods

### Nuclear single-copy gene mining strategy and data filtering

As a starting point we used the 959 APVO SSC genes of Duarte *et al.* [14]. From these, a fasta file containing the coding DNA sequences of the 959 *A. thaliana* NSCG orthologs was used as query to blast against six Cornales species available on the OneKP Project website (blastn, expectation value of 1e-5, sample IDs: QURC-*Dichroa febrifuga*, ZETY-*Hydrangea quercifolia*, BTZX-*Philadelphus inodorus*, OTAN-*Deutzia scabra*, UZNH-*Curtisia dentata* and VTLJ-*Caiophora chuquitensis*; Fig. 1). To obtain alignments for each gene ortholog, the extracted transcriptome sequences were assigned and aligned using the full length genomic sequences of the 959 *A. thaliana* NSCG as a reference (“map to reference”-tool in Geneious® version 7.1.5, Biomatters; Fig. 1). In order to select appropriate markers for primer design, the resulting 546 gene alignments were first screened for the following criteria (Fig. 1): 1) only alignments containing more than 800 bp continuously overlapping for at least four Cornales taxa were retained, 2) alignments with less than 650 bp overlapping between *H. quercifolia* and *D. febrifuga* were excluded and 3) in order to avoid potential multiple copy regions, alignments presenting scaffolds with alternative sequences for *H. quercifolia* were excluded from further steps. *Hydrangea quercifolia* is the closest relative of *H*. sect. *Cornidia* among the six Cornales species.

### Primer design, amplification and sequencing

The initial alignments contained the mined transcriptome sequences only, thus providing no information about the potential intron length and variability. For this reason, the following ten candidate nuclear loci were randomly selected from the pool for primer design: AT1G10840, AT1G63900, AT2G17975, AT3G54170, AT4G35850, AT5G12040, AT5G13030, AT5G48470, AT5G57410 and AT5G64860. These genes were subsequently aligned to the *A. thaliana* full length genomic sequence obtained from TAIR (http://www.arabidopsis.org/). Alignments were screened for regions with high abundance of non-coding regions. Primers were placed in conserved exons and designed manually.

A total of 27 primer combinations (Additional file 4) were initially tested for amplification and sequencing success on a reduced taxon set representative of the diversity within the Hydrangea I clade [sensu 44], which contains *H.* sect. *Cornidia* (see Additional file 5). Based on amplification and sequencing results, portions of three nuclear gene orthologs of *A. thaliana* (AT1G10840, AT1G63900 and AT5G57410) were chosen for testing their phylogenetic performance in a broader taxonomical sampling as described below.

### Taxon sampling

The extended taxon sampling consists of 19 accessions (Additional file 5) including 11 currently accepted *H*. sect. *Cornidia* species, as well as four undescribed species that we discovered during our field work throughout the Neotropical distribution area of the section. Additionally, *H. anomala*, which has been recovered as the sister species of *H*. sect. *Cornidia* [43–45], one species from the *H. aspera* complex and *H. quercifolia* were used as the outgroup. We rooted the phylogenetic tree with the latter species since it is positioned in a grade with *H. arborescens* and sister to the clade including the *H. aspera* complex*, H. anomala* and *H*. sect. *Cornidia* [43, 45]. Voucher information and GenBank accession numbers are provided in Additional file 5.

### Selection of plastid markers

For comparative purposes the plastid regions *rpl32–ndhF* IGS, *trnV–ndhC* IGS, *trnL–rpl32* IGS and *ndhA* intron were additionally sequenced for all species of our extended taxon sampling. These loci have previously been proposed to be the best candidates for phylogenetic studies within the tribe Hydrangeeae [45]. All plastid regions were amplified using published primers [45].

### Molecular methods

Extraction of genomic DNA was performed from fresh or silica dried leaf material using the DNeasy Plant Mini Kit (Qiagen GmbH) or a standard CTAB method [69]. PCR reactions included 8 μl dNTP (Carl Roth + Co.KG, 1.25 mM each), 10 μl Taq buffer (GoTaq® Reaction Buffer, 7.5 mM MgCl2), 2 μl of each primer (10pmol/μl), 0.25 μl of Taq DNA polymerase (GoTaq® DNA Polymerase, 5u/μl) and 0.5-2 μl of DNA template. Water was added accordingly to obtain a total volume of 50 μl. Amplification was conducted on a T3 Thermocycler (Biometra). Potential nuclear single-copy regions were amplified using a touch-up gradient PCR program (Rowther et al., 2012), including an initial denaturation at 94 °C for 2 min, a loop of 10 cycles repeated 5 times consisting on denaturation at 96 °C for 45 s, annealing at a temperature gradient (starting 5 °C below the optimal annealing temperature for each primer pair and increasing 0.5 °C every cycle) for 30 s and extension at 72 °C for 1 min/kb of expected length. A final extension at 72 °C for 7 min was applied. Plastid markers were amplified following standard protocols adopted from Samain et al. [44]. PCR products were run on a 1.2 % agarose gel and subsequently purified using a gel extraction kit (Macherey & Nagel). Sequencing was performed by Macrogen Europe sequencing services.

### Tree reconstruction

Maximum Likelihood (ML) was used for phylogenetic reconstructions with the following data partitions: 1) each individual NSCG and plastid marker, 2) all NSCG combined, 3) all plastid markers combined and 4) all NSCG and plastid markers combined. Analyses were performed with RAxML v7.2.6 [70] implementing the GTR + Γ nucleotide model as recommended in the manual, using the “rapid bootstrapping and search for the best-scoring ML tree” algorithm [71]. Bootstrap replicates were set to 1000 for all analyses. Obtained phylogenetic trees were compiled and drawn using FigTree version 1.4.0 [72].

### Estimation of phylogenetic informativeness and nodal support

We applied the phylogenetic informativeness method [48] and phylogenetic signal and noise analysis [49] in order to evaluate the performance of all the nuclear and plastid regions. All partitions were explored for per site PI [48], as well as phylogenetic signal and noise [49] using the online application PhyDesign [50]. The phylogenetic tree obtained from the total combined nuclear and plastid data matrix was considered as the most reliable phylogenetic hypothesis and used as reference tree in PhyDesign, for this we converted it to “ultrametric” with the program TreeEdit v1.0a10 [73], using the non-parametric rate smoothing method [74]. This tree was subsequently rescaled assigning branch tips to time 0 and root to time 1. This modified ultrametric tree was then uploaded along with each individual nuclear and plastid data set to the PhyDesign web application. As recommended by the PhyDesign authors, the program HyPhy [75] was used for estimating DNA sequences substitution rates. We applied the best fitting models of substitution for each partition as estimated by the FindModel tool of the HCV Database Project (http://www.hiv.lanl.gov). For this, initial trees were constructed using both Weighbor [76] and PAUP version 4.0b10 ([77]; Additional file 1). PI profiles for each individual region were contrasted with the reference ultrametric tree. Maximum per site phylogenetic informativeness (PImax) values were recorded for each region in order to determine the time at which each marker is phylogenetically most informative. Additionally, nodal support of the combined nuclear and plastid datasets were estimated by dividing the number of statistically supported clades a with BS ≥ 85 by the number of possible bipartitions (i.e. the number of terminals minus three).

### Probability of correct, incorrect and polytomous resolution across nodes

To estimate the ability of nuclear and plastid markers for resolving shallow and deeper-level divergences in *H.* section *Cornidia*, we performed a phylogenetic signal and noise analysis as implemented in the PhyDesign web application and following the analytical solution of Townsend et al. [49]. For this, the probabilities of correct, incorrect and polytomous resolution given the estimated DNA sequence substitution rates and a state space of four were calculated for the nuclear and plastid data partitions at seven individual nodes: N1 (t = 0.001, T = 0.011), N2 (t = 0.01, T = 0.1), N3 (t = 0.021, T = 0.210), N4 (t = 0.028, T = 0.284), N5 (t = 0.046, T = 0.462), N6 (t = 0.064, T = 0.640) and N7 (t = 0.1, T = 1); where t = short deep internode length and T = length of the subtending branch.

### Ethics

The present study does not involve humans, human data or animals.

## Data accessibility

GenBank accession numbers of the sequences used in the present study are provided in the additional file 5.

## Additional files

Additional file 1:
**Models of substitution and Gene Ontology annotations.** Word file listing the best fitting models of substitution for each data partition applied in the analysis of phylogenetic informativeness, as well as Gene Ontology annotations for selected NSCG used to infer phylogenetic relationships among *H*. sect. *Cornidia* species. Additional file 2:
**Regions excluded from phylogenetic analyses and informativeness, signal and noise estimations.** Excel file containing the position of excluded regions in the initial alignment and characteristics of each nuclear and plastid data matrix. Additional file 3:
**Phylogenetic trees obtained from the analyses of each data partition.** PDF file showing the phylogenetic trees obtained from the analysis of each individual nuclear and plastid region, the combined plastid data, the combined nuclear data and the total combined nuclear and plastid data sets. Additional file 4:
**Primers used for testing the ease of amplification and sequencing of 10 potential single-copy genes for** ***H***. **sect.** ***Cornidia***. Word file listing all primers that were specifically designed for this study. Non-coding coverage and spanned lengths correspond to that in *A. thaliana*. Primer combinations denoted in bold correspond to gene regions selected by their ease of amplification and sequencing. Additional file 5:
**GenBank accession numbers and voucher information.** Excel file detailing the GenBank accession numbers and voucher information associated with the extended and reduced taxon samplings.
