# Supplementary material for: A genome-scale mining strategy for recovering novel rapidly-evolving nuclear single-copy genes for addressing shallow-scale phylogenetics in Hydrangea
Source: BMC Evol Biol. 2015 Jul 4;15:132. doi: 10.1186/s12862-015-0416-z (PMC4491267; doi:10.1186/s12862-015-0416-z)
Supplement: Additional file 1: — Models of substitution and Gene Ontology annotations. Word file listing the best fitting models of substitution for each data partition applied in the analysis of phylogenetic informativeness, as well as Gene Ontology annotations for selected NSCG used to infer phylogenetic relationships among H. sect. Cornidia species. [file 12862_2015_416_MOESM1_ESM.docx]

| **Partition** | **Model of evolution** |
| --- | --- |
| TIF3H1 (AT1G10840) | TrN: Tamura-Nei plus Gamma |
| DAL1 (AT1G63900) | HKY: Hasegawa-Kishino-Yano |
| AT5G57410 | TrN: Tamura-Nei |
| *rpl32–ndhF* IGS | GTR: General Time Reversible |
| *trnV–ndhC* IGS | GTR: General Time Reversible |
| *trnL–rpl32* IGS | GTR: General Time Reversible |
| *ndhA* intron | GTR: General Time Reversible plus Gamma |

| **Partition** | **GO Biological Process** | **GO Cellular Component** |
| --- | --- | --- |
| TIF3H1 (AT1G10840) | cullin deneddylation, photomorphogenesis, translational initiation | cytoplasm, cytosol, eukaryotic translation initiation factor 3 complex |
| DAL1 (AT1G63900) | chloroplast organization, protein import into chloroplast stroma | chloroplast envelope, mitochondrion |
| AT5G57410 | response to cadmium ion | microtubule associated complex, nucleus |
