# Supplementary material for: A genome-scale mining strategy for recovering novel rapidly-evolving nuclear single-copy genes for addressing shallow-scale phylogenetics in Hydrangea
Source: BMC Evol Biol. 2015 Jul 4;15:132. doi: 10.1186/s12862-015-0416-z (PMC4491267; doi:10.1186/s12862-015-0416-z)
Supplement: Additional file 3: — Phylogenetic trees obtained from the analyses of each data partition. PDF file showing the phylogenetic trees obtained from the analysis of each individual nuclear and plastid region, the combined plastid data, the combined nuclear data and the total combined nuclear and plastid data sets. [file 12862_2015_416_MOESM3_ESM.pdf]

Total Combined

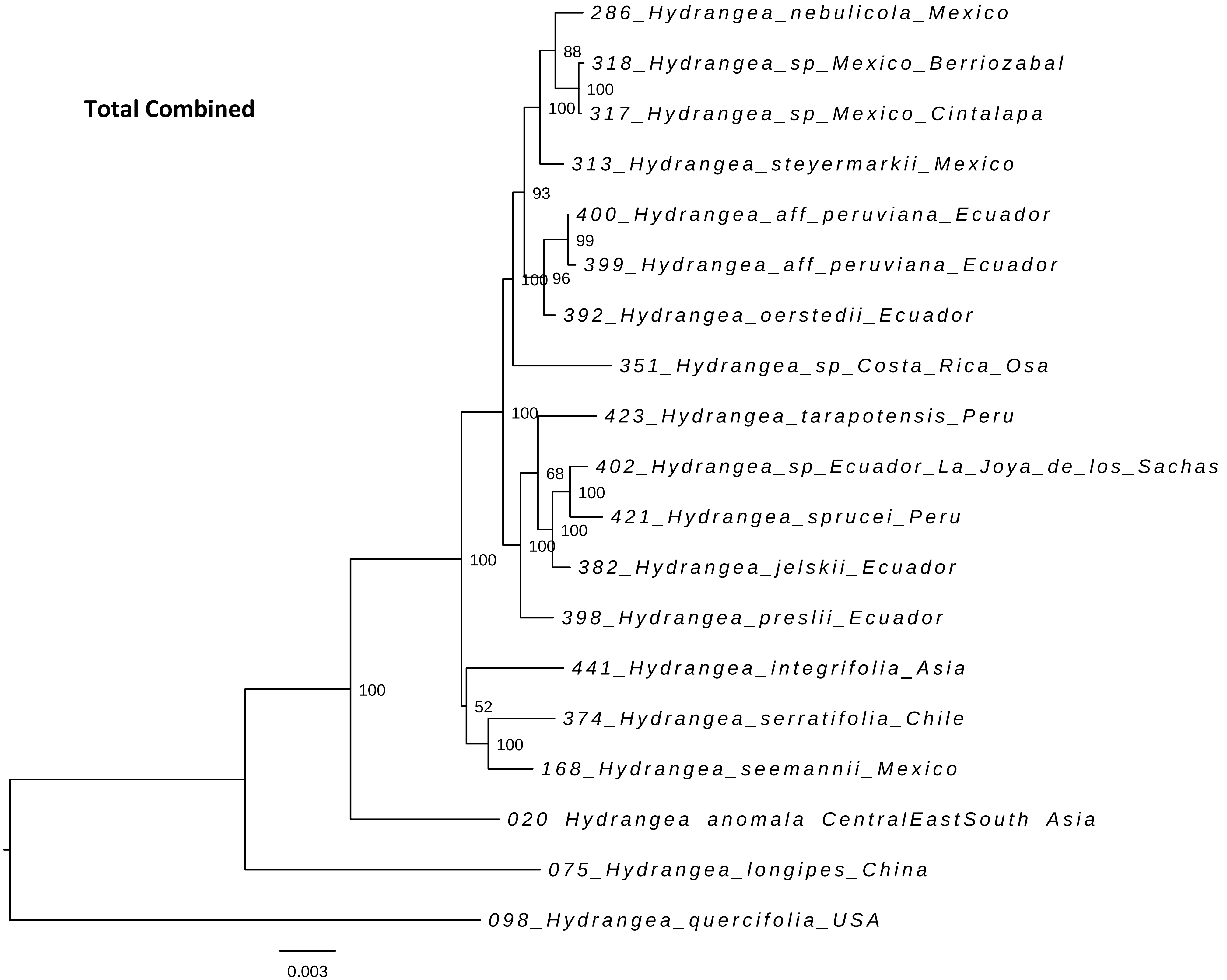

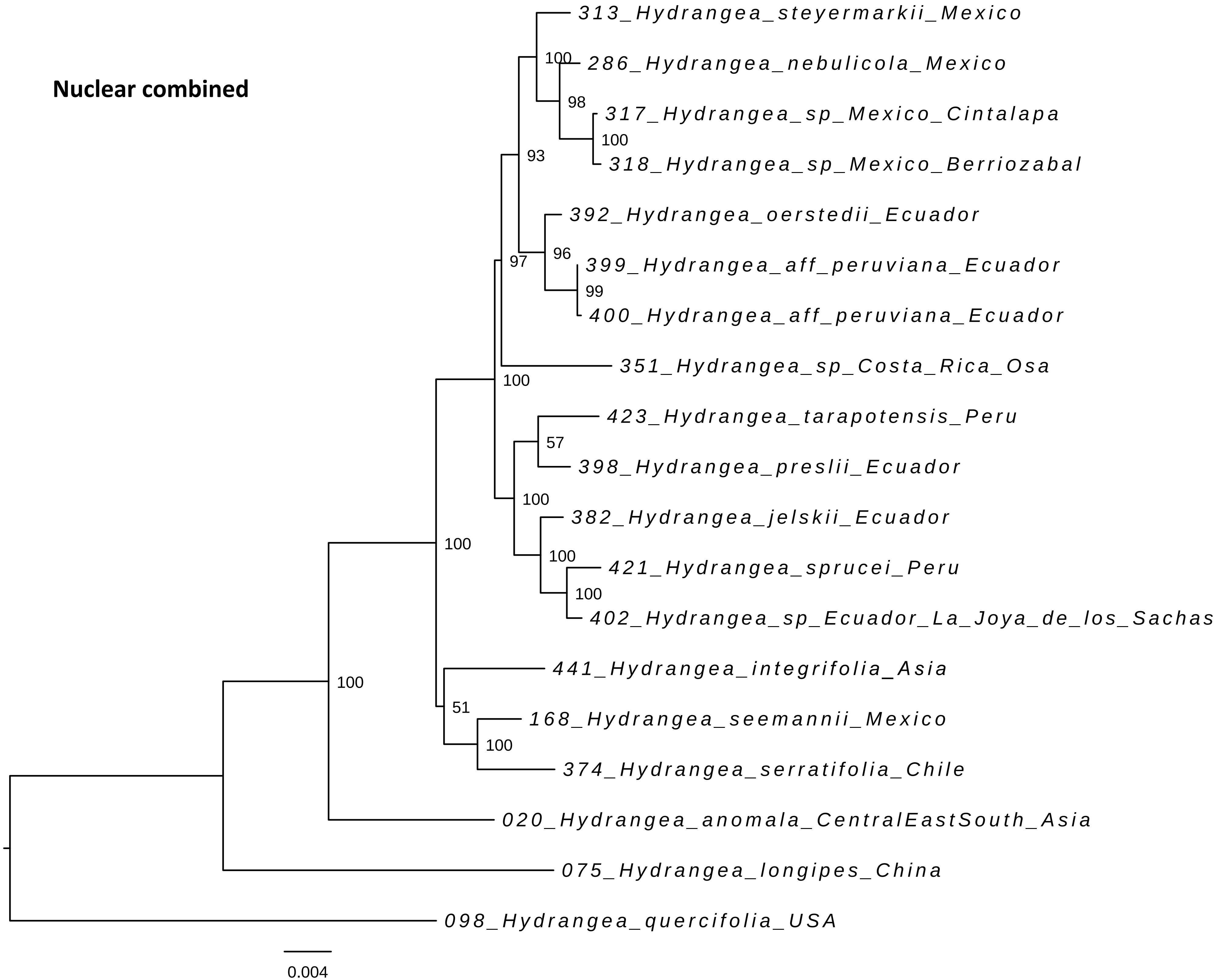
Nuclear combined
313_Hydrangea_steyermarkii_Mexico
286_Hydrangea_nebulicola_Mexico
317_Hydrangea_sp_Mexico_Cintalapa
318_Hydrangea_sp_Mexico_Berriozabal
392_Hydrangea_oerstedii_Ecuador
399_Hydrangea_aff_peruviana_Ecuador
400_Hydrangea_aff_peruviana_Ecuador
351_Hydrangea_sp_Costa_Rica_Osa
423_Hydrangea_tarapotensis_Peru
398_Hydrangea_preslii_Ecuador
382_Hydrangea_jelskii_Ecuador
421_Hydrangea_sprucei_Peru
402_Hydrangea_sp_Ecuador_La_Joya_de_los_Sachas
441_Hydrangea_integrifolia_Asia
168_Hydrangea_seemannii_Mexico
374_Hydrangea_serratifolia_Chile
020_Hydrangea_anomala_CentralEastSouth_Asia
075_Hydrangea_longipes_China
098_Hydrangea_quercifolia_USA
100
98
100
93
96
99
97
100
57
100
100
100
100
51
100
100
0.004

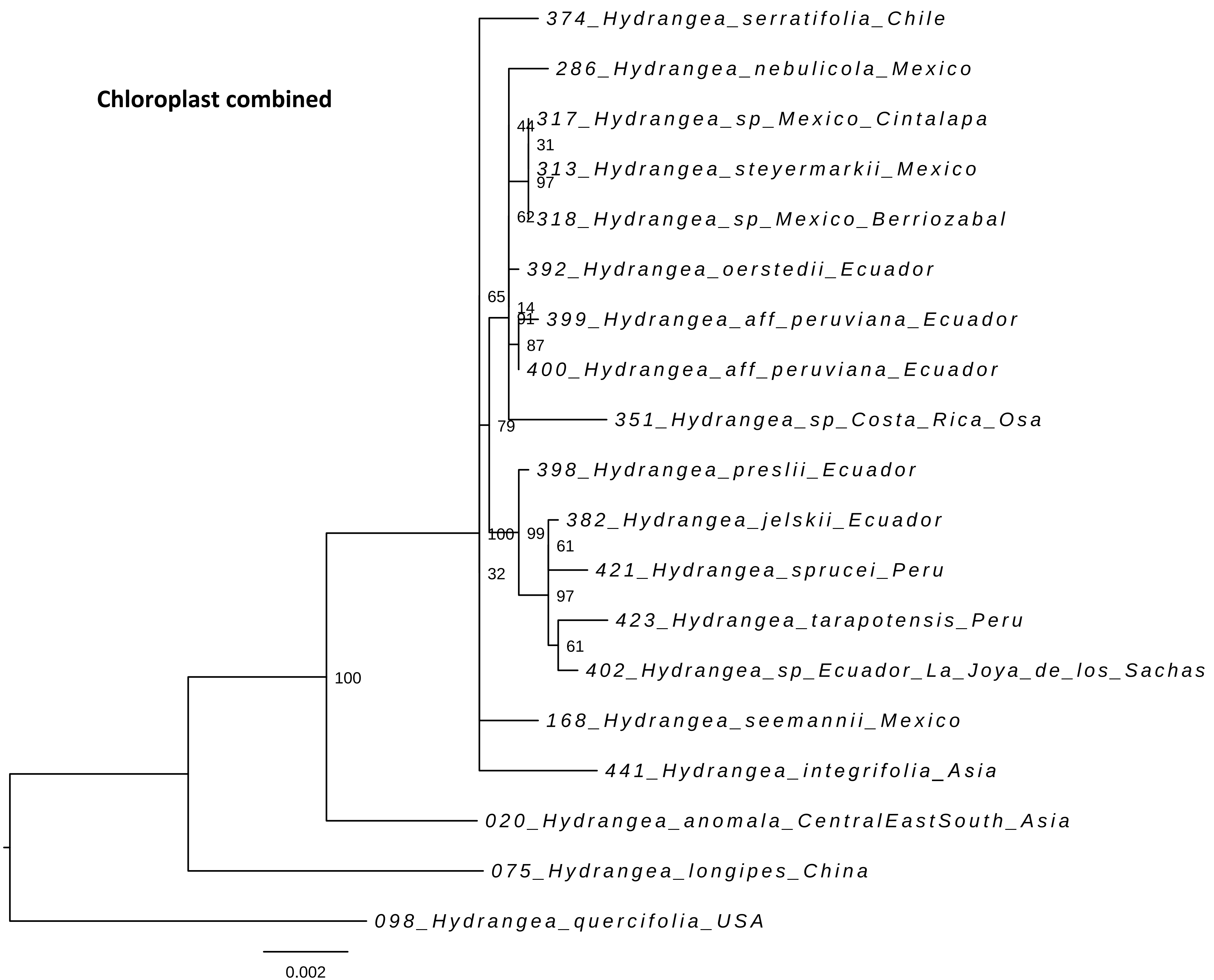
Chloroplast combined
374_Hydrangea_serratifolia_Chile
286_Hydrangea_nebulicola_Mexico
317_Hydrangea_sp_Mexico_Cintalapa
313_Hydrangea_steyermarkii_Mexico
318_Hydrangea_sp_Mexico_Berriozabal
392_Hydrangea_oerstedii_Ecuador
399_Hydrangea_aff_peruviana_Ecuador
400_Hydrangea_aff_peruviana_Ecuador
351_Hydrangea_sp_Costa_Rica_Osa
398_Hydrangea_preslii_Ecuador
382_Hydrangea_jelskii_Ecuador
421_Hydrangea_sprucei_Peru
423_Hydrangea_tarapotensis_Peru
402_Hydrangea_sp_Ecuador_La_Joya_de_los_Sachas
168_Hydrangea_seemannii_Mexico
441_Hydrangea_integrifolia_Asia
020_Hydrangea_anomala_CentralEastSouth_Asia
075_Hydrangea_longipes_China
098_Hydrangea_quercifolia_USA
44
31
97
62
65
14
91
87
79
100
99
61
32
97
61
100
0.002

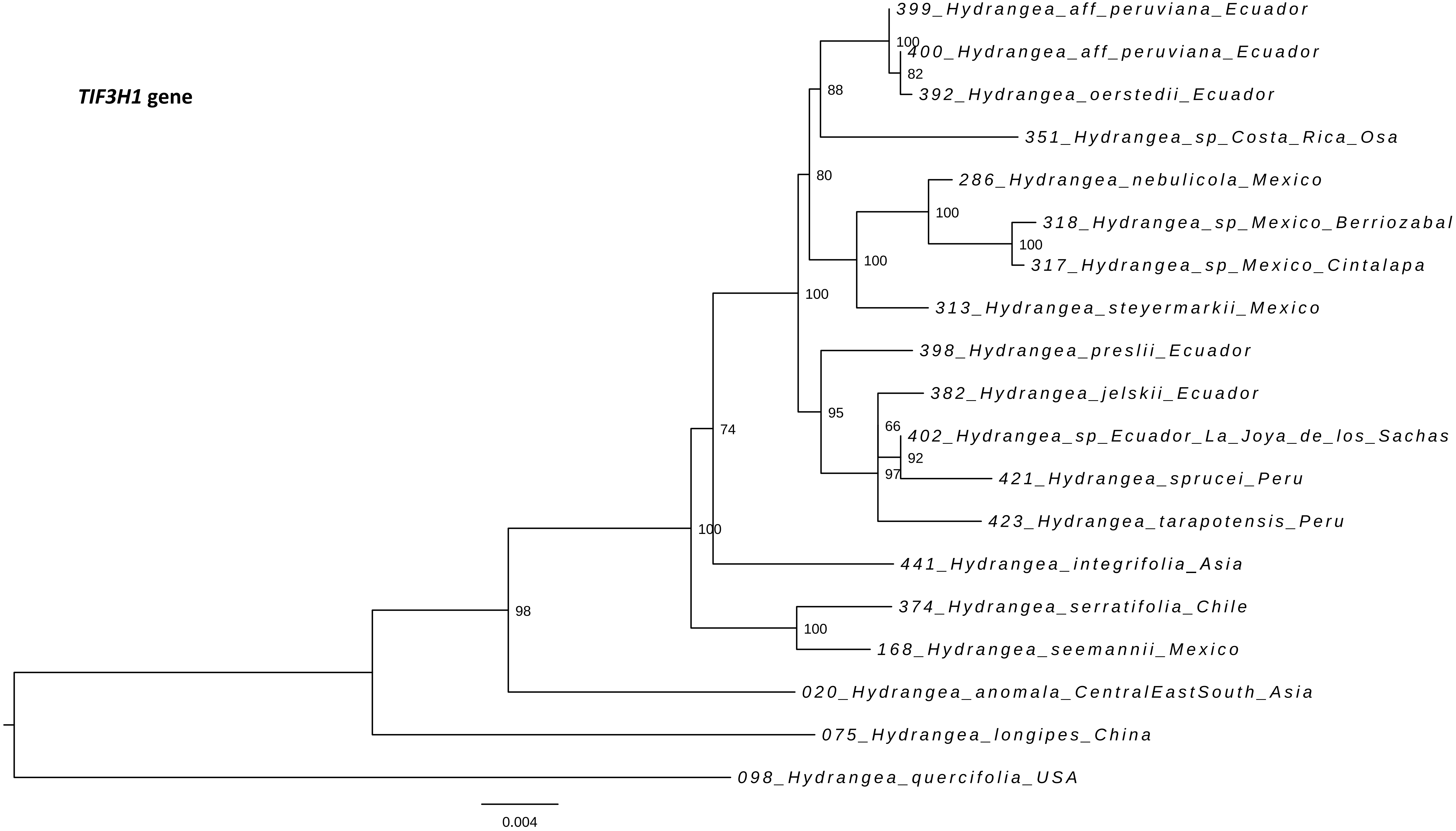

TIF3H1 gene
399_Hydrangea_aff_peruviana_Ecuador
400_Hydrangea_aff_peruviana_Ecuador
392_Hydrangea_oerstedii_Ecuador
351_Hydrangea_sp_Costa_Rica_Osa
286_Hydrangea_nebulicola_Mexico
318_Hydrangea_sp_Mexico_Berriozabal
317_Hydrangea_sp_Mexico_Cintalapa
313_Hydrangea_steyermarkii_Mexico
398_Hydrangea_preslii_Ecuador
382_Hydrangea_jelskii_Ecuador
402_Hydrangea_sp_Ecuador_La_Joya_de_los_Sachas
421_Hydrangea_sprucei_Peru
423_Hydrangea_tarapotensis_Peru
441_Hydrangea_integrifolia_Asia
374_Hydrangea_serratifolia_Chile
168_Hydrangea_seemannii_Mexico
020_Hydrangea_anomala_CentralEastSouth_Asia
075_Hydrangea_longipes_China
098_Hydrangea_quercifolia_USA
100
82
88
80
100
100
100
100
95
66
92
97
74
100
100
98
0.004

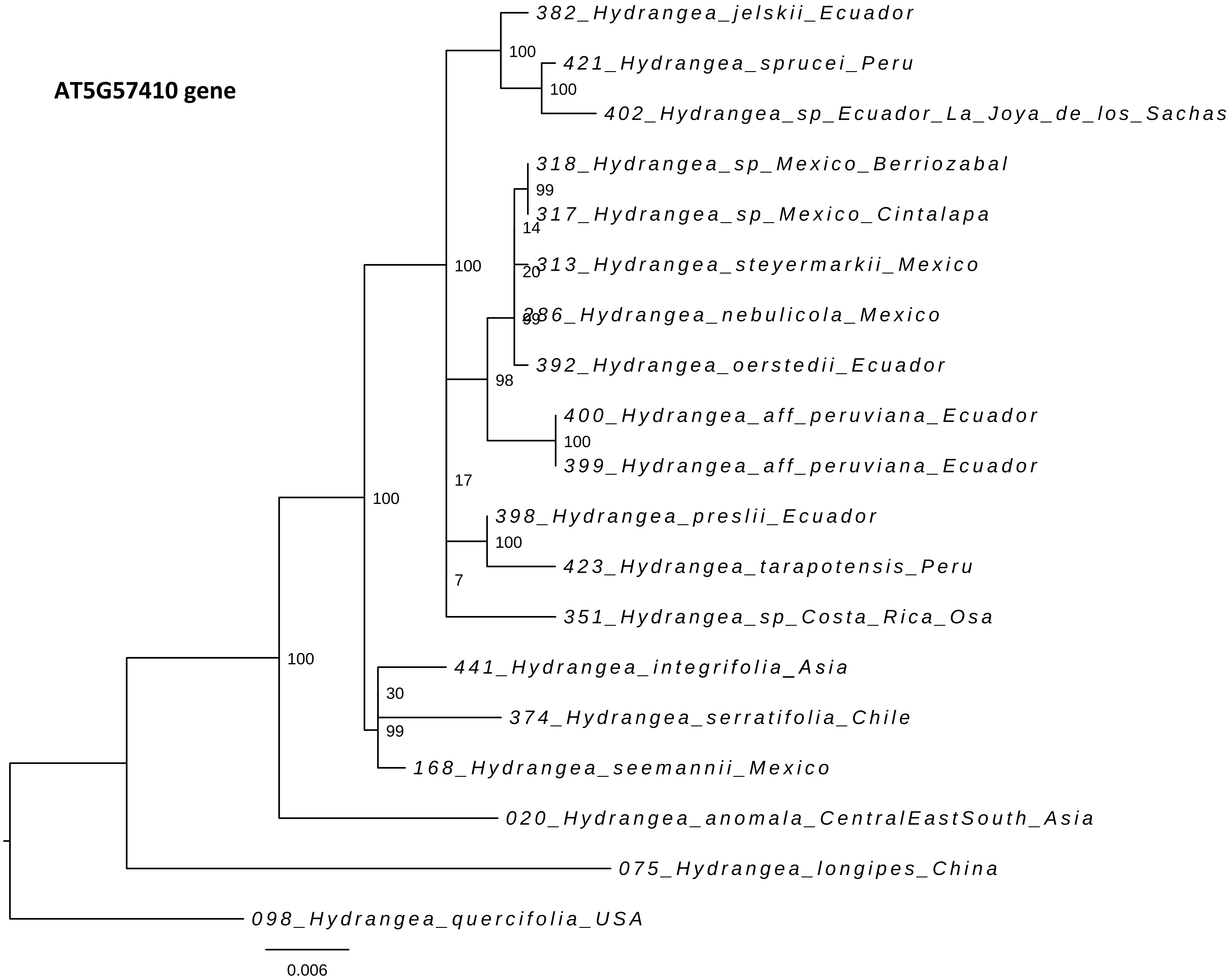
AT5G57410 gene
382_Hydrangea_jelskii_Ecuador
421_Hydrangea_sprucei_Peru
402_Hydrangea_sp_Ecuador_La_Joya_de_los_Sachas
318_Hydrangea_sp_Mexico_Berriozabal
317_Hydrangea_sp_Mexico_Cintalapa
313_Hydrangea_steyermarkii_Mexico
286_Hydrangea_nebulicola_Mexico
392_Hydrangea_oerstedii_Ecuador
400_Hydrangea_aff_peruviana_Ecuador
399_Hydrangea_aff_peruviana_Ecuador
398_Hydrangea_preslii_Ecuador
423_Hydrangea_tarapotensis_Peru
351_Hydrangea_sp_Costa_Rica_Osa
441_Hydrangea_integrifolia_Asia
374_Hydrangea_serratifolia_Chile
168_Hydrangea_seemannii_Mexico
020_Hydrangea_anomala_CentralEastSouth_Asia
075_Hydrangea_longipes_China
098_Hydrangea_quercifolia_USA
100
100
99
14
20
100
98
100
17
100
7
100
30
99
100
0.006

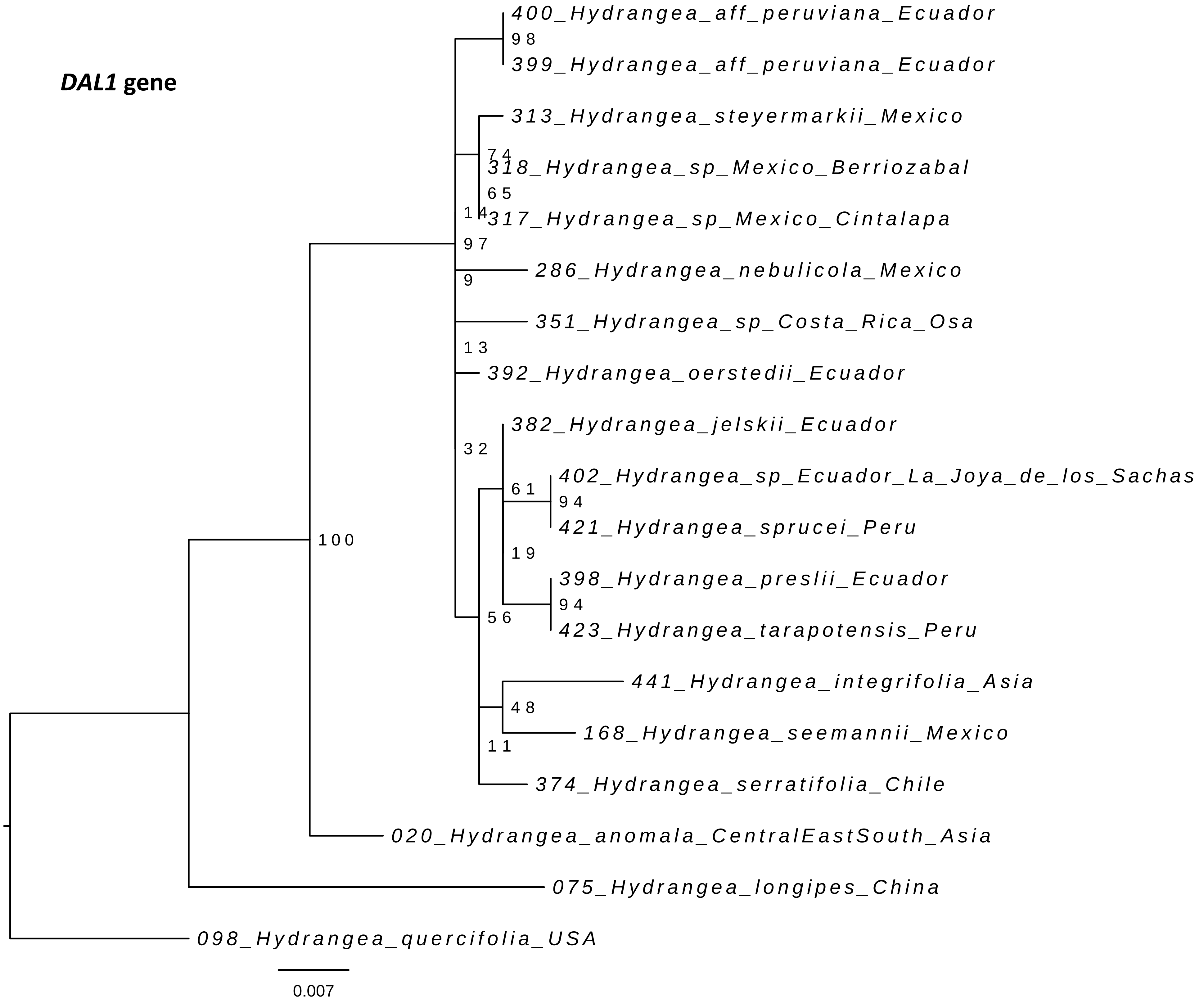
DAL1 gene
400_Hydrangea_aff_peruviana_Ecuador
98
399_Hydrangea_aff_peruviana_Ecuador
313_Hydrangea_steyermarkii_Mexico
74
318_Hydrangea_sp_Mexico_Berriozabal
65
14
317_Hydrangea_sp_Mexico_Cintalapa
97
286_Hydrangea_nebulicola_Mexico
9
351_Hydrangea_sp_Costa_Rica_Osa
13
392_Hydrangea_oerstedii_Ecuador
382_Hydrangea_jelskii_Ecuador
32
402_Hydrangea_sp_Ecuador_La_Joya_de_los_Sachas
61
94
421_Hydrangea_sprucei_Peru
100
19
398_Hydrangea_preslii_Ecuador
94
423_Hydrangea_tarapotensis_Peru
56
441_Hydrangea_integrifolia_Asia
48
168_Hydrangea_seemannii_Mexico
11
374_Hydrangea_serratifolia_Chile
020_Hydrangea_anomala_CentralEastSouth_Asia
075_Hydrangea_longipes_China
098_Hydrangea_quercifolia_USA
0.007

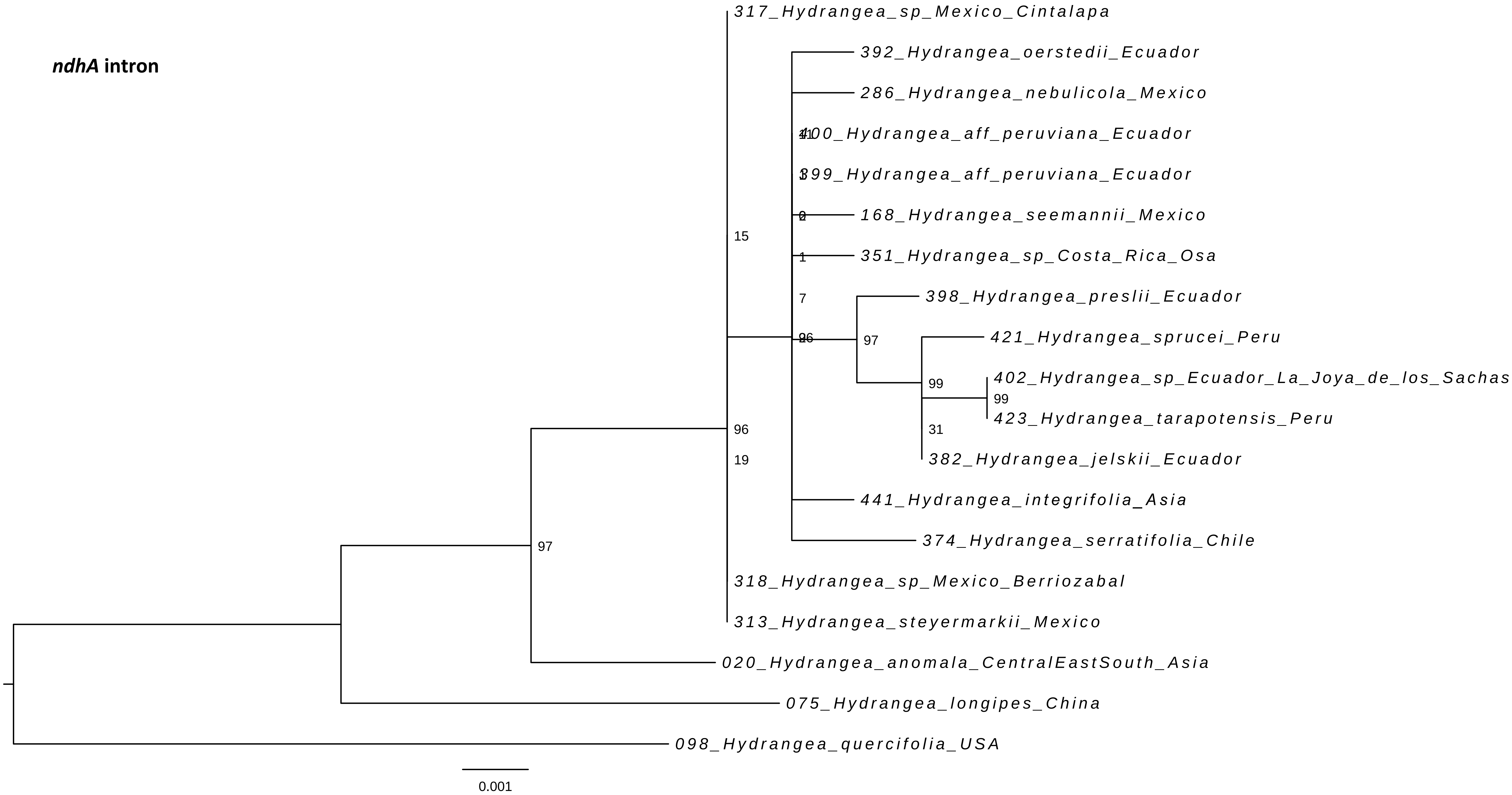

ndhA intron
317_Hydrangea_sp_Mexico_Cintalapa
392_Hydrangea_oerstedii_Ecuador
286_Hydrangea_nebulicola_Mexico
400_Hydrangea_aff_peruviana_Ecuador
399_Hydrangea_aff_peruviana_Ecuador
168_Hydrangea_seemannii_Mexico
351_Hydrangea_sp_Costa_Rica_Osa
398_Hydrangea_preslii_Ecuador
421_Hydrangea_sprucei_Peru
402_Hydrangea_sp_Ecuador_La_Joya_de_los_Sachas
423_Hydrangea_tarapotensis_Peru
382_Hydrangea_jelskii_Ecuador
441_Hydrangea_integrifolia_Asia
374_Hydrangea_serratifolia_Chile
318_Hydrangea_sp_Mexico_Berriozabal
313_Hydrangea_steyermarkii_Mexico
020_Hydrangea_anomala_CentralEastSouth_Asia
075_Hydrangea_longipes_China
098_Hydrangea_quercifolia_USA
15
1
7
97
99
99
31
96
19
97
0.001

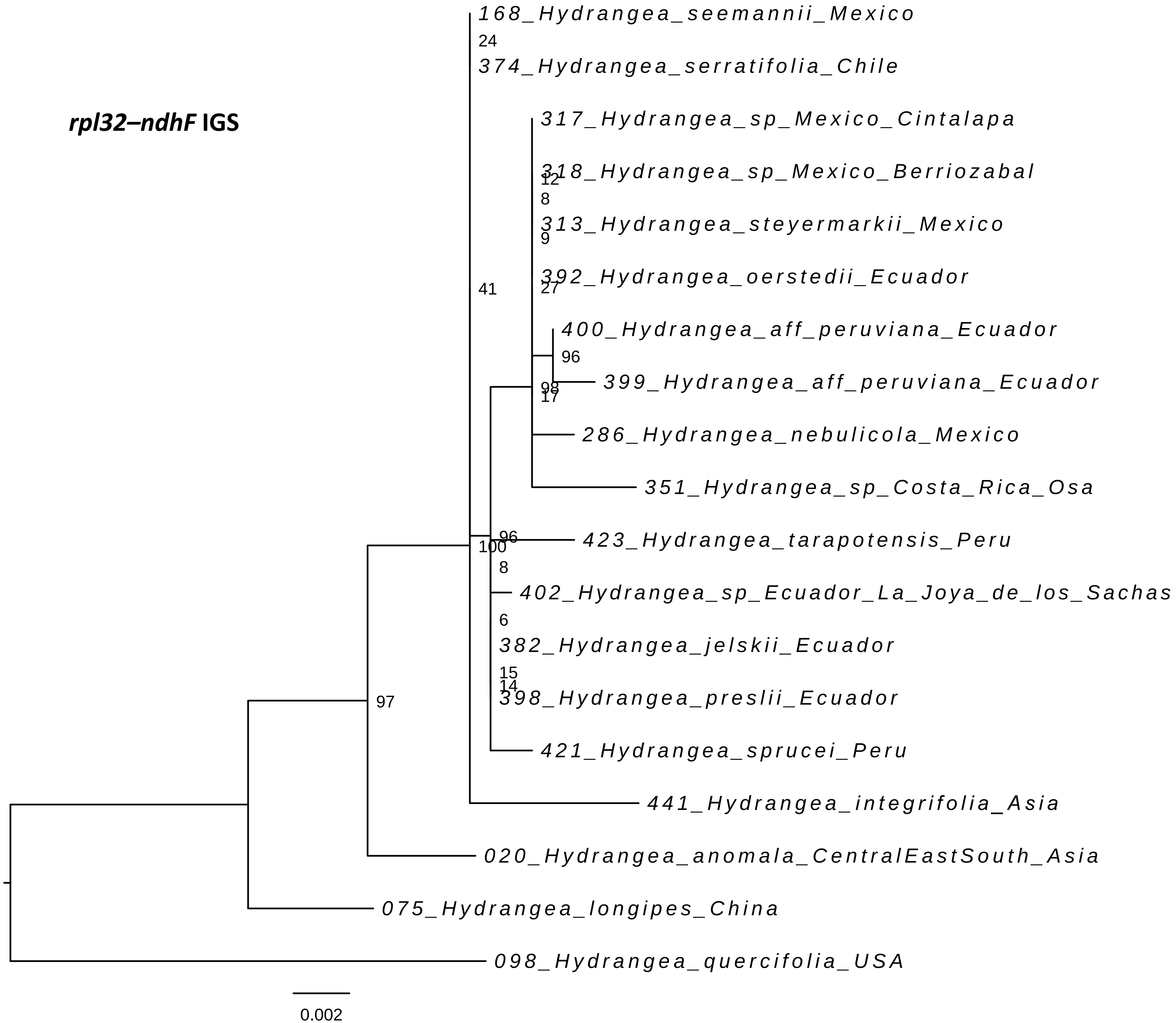
rpl32–ndhF IGS
168_Hydrangea_seemannii_Mexico
24
374_Hydrangea_serratifolia_Chile
317_Hydrangea_sp_Mexico_Cintalapa
318_Hydrangea_sp_Mexico_Berriozabal
12
8
313_Hydrangea_steyermarkii_Mexico
9
392_Hydrangea_oerstedii_Ecuador
41
27
400_Hydrangea_aff_peruviana_Ecuador
96
399_Hydrangea_aff_peruviana_Ecuador
98
17
286_Hydrangea_nebulicola_Mexico
351_Hydrangea_sp_Costa_Rica_Osa
96
423_Hydrangea_tarapotensis_Peru
100
8
402_Hydrangea_sp_Ecuador_La_Joya_de_los_Sachas
6
382_Hydrangea_jelskii_Ecuador
15
14
398_Hydrangea_preslii_Ecuador
97
421_Hydrangea_sprucei_Peru
441_Hydrangea_integrifolia_Asia
020_Hydrangea_anomala_CentralEastSouth_Asia
075_Hydrangea_longipes_China
098_Hydrangea_quercifolia_USA
0.002

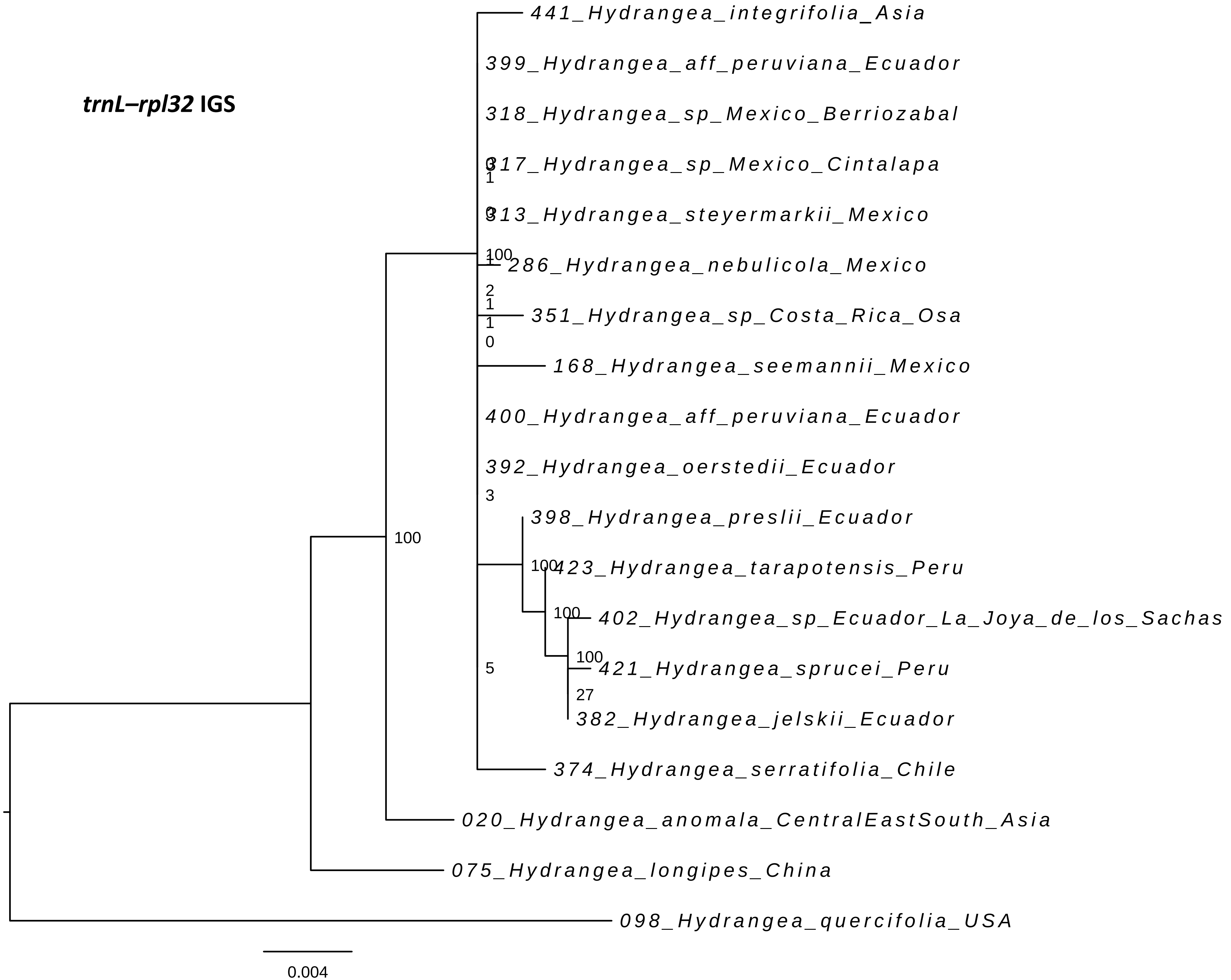

trnL–rpl32 IGS
441_Hydrangea_integrifolia_Asia
399_Hydrangea_aff_peruviana_Ecuador
318_Hydrangea_sp_Mexico_Berriozabal
317_Hydrangea_sp_Mexico_Cintalapa
313_Hydrangea_steyermarkii_Mexico
286_Hydrangea_nebulicola_Mexico
351_Hydrangea_sp_Costa_Rica_Osa
168_Hydrangea_seemannii_Mexico
400_Hydrangea_aff_peruviana_Ecuador
392_Hydrangea_oerstedii_Ecuador
398_Hydrangea_preslii_Ecuador
423_Hydrangea_tarapotensis_Peru
402_Hydrangea_sp_Ecuador_La_Joya_de_los_Sachas
421_Hydrangea_sprucei_Peru
382_Hydrangea_jelskii_Ecuador
374_Hydrangea_serratifolia_Chile
020_Hydrangea_anomala_CentralEastSouth_Asia
075_Hydrangea_longipes_China
098_Hydrangea_quercifolia_USA
100
100
100
100
100
27
0.004

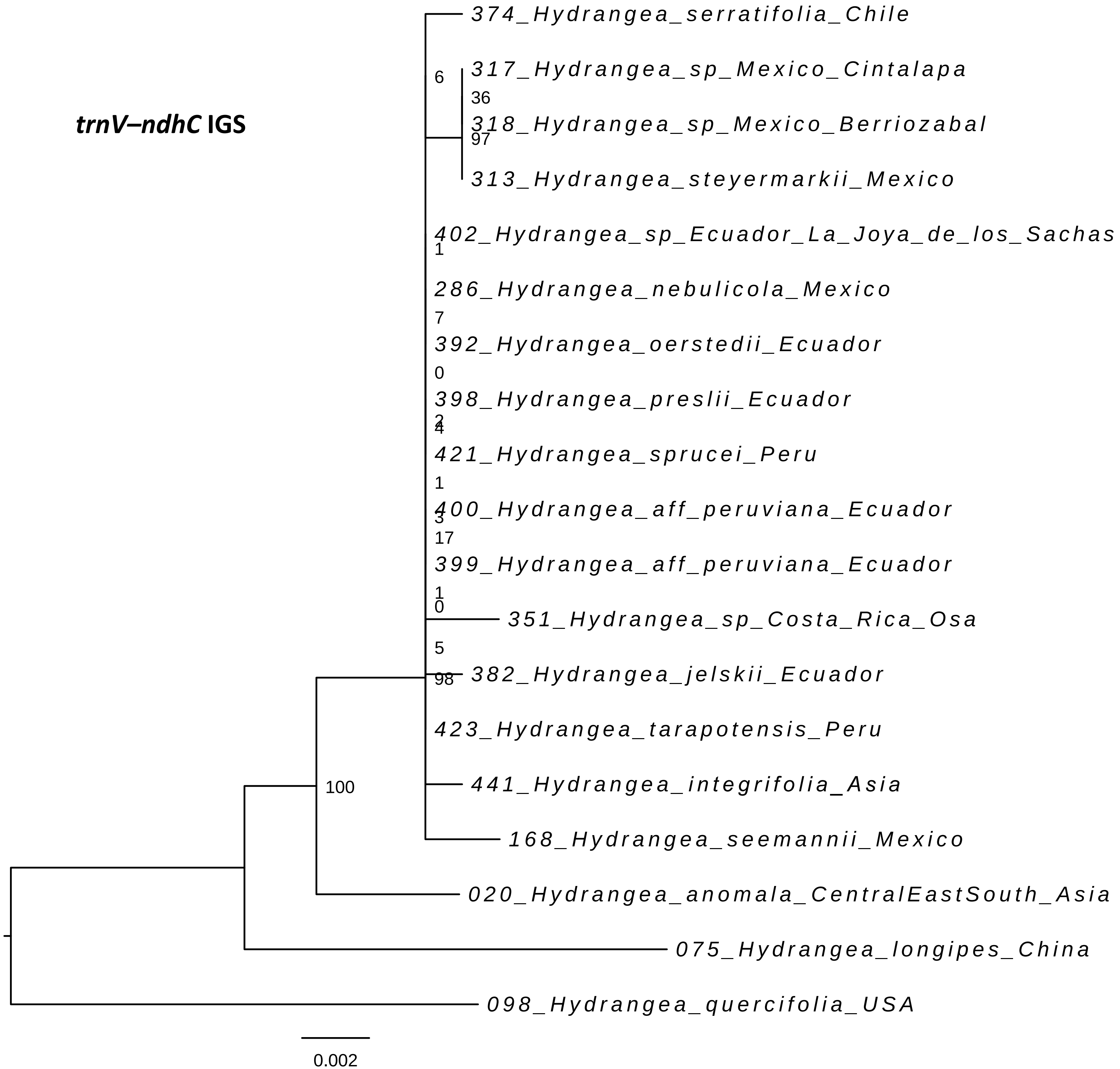

trnV–ndhC IGS
374_Hydrangea_serratifolia_Chile
317_Hydrangea_sp_Mexico_Cintalapa
318_Hydrangea_sp_Mexico_Berriozabal
313_Hydrangea_steyermarkii_Mexico
402_Hydrangea_sp_Ecuador_La_Joya_de_los_Sachas
286_Hydrangea_nebulicola_Mexico
392_Hydrangea_oerstedii_Ecuador
398_Hydrangea_preslii_Ecuador
421_Hydrangea_sprucei_Peru
400_Hydrangea_aff_peruviana_Ecuador
399_Hydrangea_aff_peruviana_Ecuador
351_Hydrangea_sp_Costa_Rica_Osa
382_Hydrangea_jelskii_Ecuador
423_Hydrangea_tarapotensis_Peru
441_Hydrangea_integrifolia_Asia
168_Hydrangea_seemannii_Mexico
020_Hydrangea_anomala_CentralEastSouth_Asia
075_Hydrangea_longipes_China
098_Hydrangea_quercifolia_USA
6
36
97
1
7
0
2
4
1
3
17
1
0
5
98
100
0.002
