# Supplementary material for: A genome-scale mining strategy for recovering novel rapidly-evolving nuclear single-copy genes for addressing shallow-scale phylogenetics in Hydrangea
Source: BMC Evol Biol. 2015 Jul 4;15:132. doi: 10.1186/s12862-015-0416-z (PMC4491267; doi:10.1186/s12862-015-0416-z)
Supplement: Additional file 4: — Primers used for testing the ease of amplification and sequencing of 10 potential single-copy genes for H. sect. Cornidia. Word file listing all primers that were specifically designed for this study. Non-coding coverage and spanned lengths correspond to that in A. thaliana. Primer combinations denoted in bold correspond to gene regions selected by their ease of amplification and sequencing. [file 12862_2015_416_MOESM4_ESM.docx]

Additional file 1. Primers used for testing the ease of amplification and sequencing of 10 potential single-copy genes for *H*. sect. *Cornidia*. All listed primers were specifically designed for this study. Non-coding coverage and spanned lengths correspond to that in *A. thaliana*. Primer combinations denoted in bold correspond to gene regions selected by their ease of amplification and sequencing.

| ***A. thaliana* ortholog** | **F-primer name: sequence 5´–3´** | **R-primer name: sequence 5´–3´** | **Non-coding coverage (bp)** | **Spanned length (bp)** |
| --- | --- | --- | --- | --- |
| **AT1G10840 (*TIF3H1*)** | H-AT1G10840-1F: TTCTTGGGTTRGATGTMGGCAG | AT1G10840-1R: ATTTCCTCAAAGATATCCACCCAT | 543 | 949 |
|  | **H-AT1G10840-2F: TTCAAGGTCCAACCAAGGTGTCTTA** | **H-AT1G10840-2R: TTGCTGCTGGGCTTGCTGAC** | **396** | **763** |
|  | **H-AT1G10840-2F: TTCAAGGTCCAACCAAGGTGTCTTA** | **H-AT1G10840-3R: TGATTTGTTATGAGAAAACTATCC** | **581** | **1091** |
|  | **H-AT1G10840-3F: CAACTATCAACCAATCCATT** | **H-AT1G10840-3R: TGATTTGTTATGAGAAAACTATCC** | **270** | **545** |
| **AT1G63900 (*DAL1*)** | **H-AT1G63900-1F: GGAGGTTCTCAAATCGGTTACT** | **H-AT1G63900-2R: TCCCAGTTGGAAGAASCCGTT** | **340** | **789** |
|  | **H-AT1G63900-1F: GGAGGTTCTCAAATCGGTTACT** | **H-AT1G63900-1R: TTTGTGAGAAACATAGAAAGGC** | **562** | **1106** |
|  | **H-AT1G63900-2F: ATTTCTGGAAGAGTTGGTTCAG** | **H-AT1G63900-1R: TTTGTGAGAAACATAGAAAGGC** | **480** | **930** |
| AT2G17975 | H-AT2G17975-1F: CCAAAGGAGRTAGCAGCAATGCC | H-AT2G17975-1R: GATTCCCACCAGAACCTCCCATT | 286 | 659 |
| AT3G54170 | H-AT3G54170-1F: AAGTAGAAGAAACTGCACCTGGT | H-AT3G54170-1R: CGCTCTCAAGTCCCGAACTGC | 491 | 832 |
|  | H-AT3G54170-2F: TGGTGGTCASTTWTCTTCAGACTCTAA | H-AT3G54170-3R: CATTTAGCCATTAGCATCTTCC | 861 | 1204 |
|  | H-AT3G54170-3F: GCAGTTCGGGACTTGAGAGCG | H-AT3G54170-3R: CATTTAGCCATTAGCATCTTCC | 651 | 857 |
|  | H-AT3G54170-3F: GCAGTTCGGGACTTGAGAGCG | H-AT3G54170-4R: TGACTTCTGAGTTCCGCATTCTG | 796 | 1119 |
| AT4G35850 | H-AT4G35850-1F: GCACTGAATTGGAGTACAACAA | H-AT4G35850-2R: TATTGGTGTCTTRTTCTTATGTGC | 645 | 1132 |
|  | H-AT4G35850-2F: AACCAGWGCGAGATACATTCCAC | H-AT4G35850-2R: TATTGGTGTCTTRTTCTTATGTGC | 473 | 849 |
| AT5G12040 | H-AT5G12040-1F: TCAGTAACGGAGGACAAGGARAG | H-AT5G12040-1R: GTCCACAATGGTAGGAGTCT | 472 | 880 |
|  | H-AT5G12040-1F: TCAGTAACGGAGGACAAGGARAG | H-AT5G12040-2R: AGTTCCTGAAACCGAATGTCATAACA | 829 | 1297 |
|  | H-AT5G12040-2F: CAGTTTATGCTGAGGACATTGATG | H-AT5G12040-2R: AGTTCCTGAAACCGAATGTCATAACA | 680 | 1018 |
|  | H-AT5G12040-3F: AGACTCCTACCATTGTGGAC | H-AT5G12040-3R: AAGCCACGTAACCAGCACCATCA | 773 | 1022 |
| AT5G13030 | H-AT5G13030-1F: TCGTTTGTTCGGGASTTRCCT | AT5G13030-1R: CTCCAACCAAAGGYGATGCC | 611 | 831 |
|  | H-AT5G13030-1F: TCGTTTGTTCGGGASTTRCCT | H-AT5G13030-2R: TGCCCGACCATCRCCCAACT | 700 | 995 |
| AT5G48470 | H-AT5G48470-1F: ATCATCAAGGAGACYGTWTTATCC | H-AT5G48470-1R: CTGTGATGCCAGAGACATTTCCA | 485 | 1119 |
|  | H-AT5G48470-2F: CCCCAGAARGAAAGYAGACA | H-AT5G48470-1R: CTGTGATGCCAGAGACATTTCCA | 388 | 943 |
| **AT5G57410** | **H-AT5G57410-1F: CGATGTTACAAACTTGGACCATTG** | **H-AT5G57410-1R: TCCTTTTCTTTTTTCTTCAT** | **416** | **846** |
|  | **H-AT5G57410-1F: CGATGTTACAAACTTGGACCATTG** | **H-AT5G57410-2R: AAGTAAATTCATTATCTCCATGCCTGA** | **497** | **1015** |
|  | **H-AT5G57410-2F: TGCTAATGAACAAAGACAAA** | **H-AT5G57410-2R: AAGTAAATTCATTATCTCCATGCCTGA** | **424** | **767** |
| AT5G64860 | H-AT5G64860-1F: CAACAGTTCYTATTCCAAMGGCAATGG | H-AT5G64860-1R: CTTTCCAACTGCTCTGAAGATGGCA | 511 | 952 |
|  | H-AT5G64860-3F: ATCAGTATTATGGGAGACAT | H-AT5G64860-1R: CTTTCCAACTGCTCTGAAGATGGCA | 511 | 892 |
